# Associations between maternal metabolic conditions and neurodevelopmental conditions in offspring: the mediating effects of obstetric and neonatal complications

**DOI:** 10.1186/s12916-023-03116-x

**Published:** 2023-11-07

**Authors:** Shuyun Chen, Xi Wang, Brian K. Lee, Renee M. Gardner

**Affiliations:** 1 Department of Global Public Health, Karolinska Institutet, Stockholm, Sweden; 2 PolicyLab, Children’s Hospital of Philadelphia, Philadelphia, PA USA; 3 Department of Epidemiology and Biostatistics, Drexel University School of Public Health, Philadelphia, PA USA; 4 A.J. Drexel Autism Institute, Philadelphia, PA USA

**Keywords:** Autism, Intellectual disability, Attention deficit, Hyperactivity disorder, Pregestational diabetes, Gestational diabetes, Overweight, Obesity, Obstetric complications, Neonatal complications

## Abstract

**Background:**

Maternal pre-gestational diabetes (PGDM), gestational diabetes mellitus (GDM), and overweight/obesity have been associated with increased risks of offspring neurodevelopmental conditions (NDCs) including autism, intellectual disability (ID), and attention deficit/hyperactivity disorder (ADHD). Less is known about whether and how obstetric and neonatal complications (e.g., preterm birth, neonatal asphyxia) could mediate these associations.

**Methods:**

In this Swedish register-based cohort study, we examined complications during pregnancy, delivery, and the neonatal period as potential mediators of the relationships between maternal metabolic conditions and offspring NDCs. We quantified the extent to which these obstetric and neonatal factors could mediate the associations of maternal metabolic conditions with offspring NDCs by applying parametric regression models for single mediation analyses and weighting-based methods for multiple mediation analyses under counterfactual frameworks.

**Results:**

The study sample included 2,352,969 singleton children born to 1,299,692 mothers from 1987–2010 who were followed up until December 31, 2016, of whom 135,832 children (5.8%) were diagnosed with at least one NDC. A substantial portion of the association between maternal PGDM and children’s odds of NDCs could be explained by the combined group of obstetric and neonatal complications in the multiple mediation analysis. For instance, these complications explained 44.4% of the relationship between maternal PGDM and offspring ID risk. The proportion of the relationship between maternal overweight/obesity and children’s risk of NDCs that could be explained by obstetric and neonatal complications was considerably smaller, ranging from 1.5 to 8.1%. Some complications considered on their own, including pregnancy hypertensive diseases, preterm birth, neonatal asphyxia, and hematological comorbidities, could explain at least 10% of the associations between maternal PGDM and offspring NDCs. Complications during the neonatal period showed a stronger joint mediating effect for the relationship between PGDM and offspring NDCs than those during pregnancy or delivery.

**Conclusions:**

Obstetric and neonatal complications could explain nearly half of the association between maternal PGDM and offspring risk of NDCs. The mediating effects were more pronounced for complications during the neonatal period and for specific complications such as pregnancy hypertensive diseases, preterm birth, neonatal asphyxia, and hematological comorbidities. Effective preventive strategies for offspring NDCs should holistically address both the primary metabolic issues related to PGDM and the wide array of potential complications, especially those in the neonatal period.

**Graphical Abstract:**

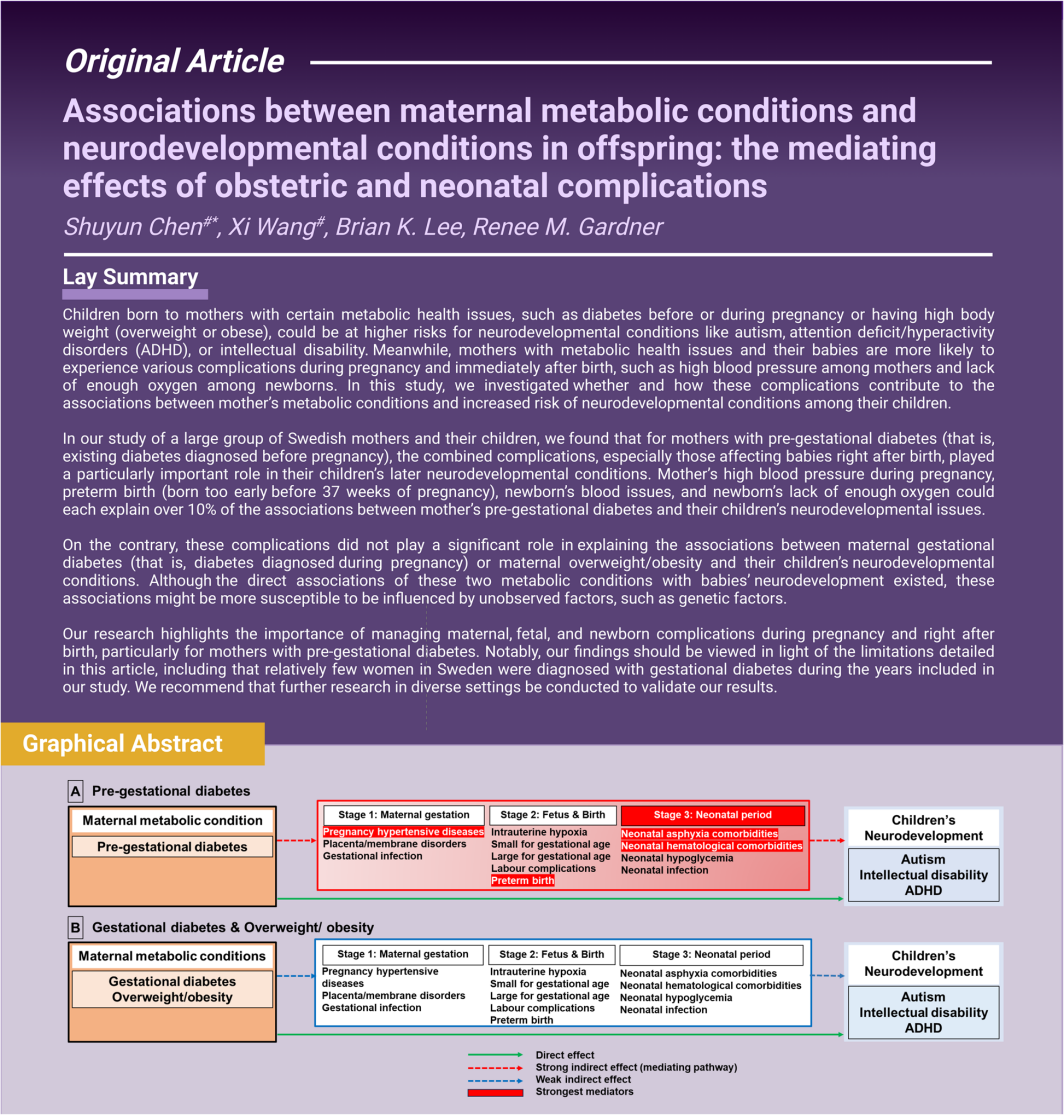

**Supplementary Information:**

The online version contains supplementary material available at 10.1186/s12916-023-03116-x.

## Background

Globally, there is an increasing trend of type 1 diabetes (T1DM), type 2 diabetes (T2DM), and gestational diabetes mellitus (GDM), which has paralleled the increase in obesity over the past few decades [1, 2]. Attention-deficit/hyperactivity disorder (ADHD) is the most frequently diagnosed neurodevelopmental condition (NDC) in childhood [3], and the prevalence of autism is on the rise [4]. Both conditions often coexist with intellectual disability (ID) [3, 4]. However, a thorough understanding of their etiologies is yet to be achieved, meaning treatment and prevention strategies are still under development. Previous studies showed that children exposed to adverse maternal metabolic conditions in utero have increased risks of NDCs, including autism, ID, and ADHD [5, 6]. While these associations may be attributed wholly or partially, to shared genetic liabilities between metabolic conditions and NDCs [7–9], other mechanisms may also explain the relationships between maternal metabolic conditions and offspring NDCs [5, 6, 10]. In particular, maternal metabolic conditions, especially pre-gestational and gestational diabetes, have been found to be associated with several obstetric and neonatal complications during pregnancy, at delivery, and after birth. These complications include preeclampsia/eclampsia [11], placenta and membrane abnormalities [12], gestational infection [13, 14], intrauterine hypoxia [15, 16], neonatal asphyxia-related comorbidities [11, 17], preterm birth [11, 17], fetal overgrowth [11, 17, 18], fetal growth impairment [19, 20], labor complications [21], neonatal hypoglycemia [22], neonatal anemia [23], neonatal polycythemia [24], neonatal jaundice [25], and neonatal infection [26]. These obstetric and neonatal complications are also associated with an increased risk of NDCs in offspring [27–31]. An unresolved question concerns the degree to which the relationship between maternal metabolic conditions and NDCs in offspring can be ascribed to obstetric and neonatal complications. In addition, given that fetal brain development progresses in a rapid and sequential pattern [32], the effects of complications in early life may differ by the timing of exposure (during pregnancy, birth, or neonatal period) [33].

In this nationwide register-based cohort study, by applying a mediation analysis approach under assumptions of a counterfactual framework, we aimed to quantify the extent to which obstetric and neonatal complications mediate the associations between maternal metabolic conditions (diabetes and adiposity) and offspring NDCs (autism, ID, and ADHD).

## Methods

### Study design and population

**Fig. 1:**
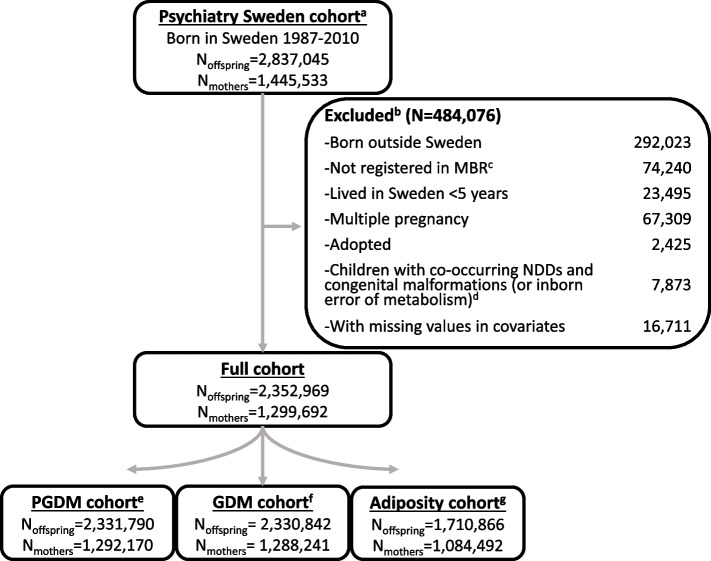
Study sample derivation. Superscript lowercase letter a “^a^” indicates the following: children born before 1987 were excluded from the study population because data on gestational diabetes mellitus (GDM) diagnoses were only available from 1987 onwards. Superscript lowercase letter b “^b^” indicates the following: exclusions were made stepwise, as illustrated. Superscript lowercase letter c “^c^” indicates the following: individuals lacking information from the Medical Birth Register (MBR) were excluded. Superscript lowercase letter d “^d^” indicates the following: children with co-morbid congenital malformations (or inborn errors of metabolism) and neurodevelopmental disorders (NDCs) were excluded, as these NDCs may be attributable to the congenital condition. Superscript lowercase letter e “^e^” indicates the following: children of mothers diagnosed with GDM were excluded from the PGDM cohort. Superscript lowercase letter f “^f^” indicates the following: children of mothers diagnosed with PGDM were excluded from the GDM cohort. Superscript lowercase letter g “^g^” indicates the following: children of mothers who were underweight or lacked BMI information were excluded from the adiposity cohort

This population-based cohort study used data from “Psychiatry Sweden,” a combination of registers for investigating the occurrence, causes, and consequences of psychiatric disorders. A detailed description is presented elsewhere [34]. All children were linked to their birth mothers and fathers via the Multi-Generation Register using the unique national identification number, which they received at birth (or on their arrival to Sweden for immigrants) [35]. We included children born from January 1, 1987, to December 31, 2010 (*n* = 2,837,045) and followed up until December 31, 2016. We excluded children who met any of the following exclusion criteria: not born in Sweden or having lived in Sweden for less than 5 years by December 31, 2016; without records in the Medical Birth Registry (MBR); multiple births (twins, triplets, etc.), as multiple pregnancies may carry more perinatal complications [36]; those who were adopted; or those with both congenital malformations and NDCs, as these co-occurring conditions could be due to genetic factors [37] (Fig. 1). We further excluded those with missing information on any of the potential confounders (0.6% of the otherwise eligible sample), with an assumption of missing at random supported by our previous work [34]. Our final sample consisted of 2,352,969 children born to 1,299,692 mothers. Ethical approval was obtained from the Stockholm regional ethical review committee (DNR 2010/1185–31/5, 2016/987–32), and informed consent was not required as the data were pseudonymized.

### Outcomes

International Classification of Diseases (ICD) codes, versions 9th and 10th (ICD-9 and ICD-10), were used for the identification of autism, ADHD, and ID cases from the Swedish National Patient Register (Additional file 1: Table S1) [38, 39]. We used the Prescription Drug Register to identify additional ADHD cases if ADHD medication was prescribed [40]. We additionally generated a variable of “Any NDCs” to represent those with any autism, ADHD, or ID diagnoses. Note that diagnoses of autism, ID, and ADHD were not mutually exclusive.

### Exposures

#### Maternal diabetes

Pre-gestational T1DM, T2DM, or GDM were identified from the National Patient Register (NPR) and MBR (Additional file 1: Table S1). A detailed description has been presented elsewhere [34]. If a mother’s diabetes diagnosis could not be clearly established as T1DM or T2DM, given the lack of distinction of these two diagnoses before ICD-10, the diagnosis was categorized as non-specified pre-gestational diabetes mellitus (PGDM-NOS). Due to the small number of cases in T2DM and PGDM-NOS which provided insufficient power for further mediation analysis on their own, we combined T1DM, T2DM, and PGDM-NOS as pre-gestational diabetes (PGDM). Within PGDM, T1DM accounted for 78.22% of identified cases.

#### Maternal adiposity

Individual body mass index (BMI) was calculated by weight (kg)/height^2^ (m^2^) measured at first antenatal care, and BMI ≥ 25 kg/m^2^ was identified as overweight or obese. We used maternal overweight or obesity status at the first antenatal visit as a proxy for high maternal adiposity. In Sweden, the initial antenatal care typically occurs around the 9–10th week of gestation, though information on date at first antenatal visit was not recorded in the MBR until 1998 [7, 41, 42]. Throughout the manuscript, we refer to maternal overweight/obesity at the first antenatal visit as high maternal adiposity. Among 2,352,969 offspring in the study population, 75.14% had information for maternal BMI. Among those with maternal BMI information, 3.23% had mothers who were underweight and were excluded from further mediation analysis. An adiposity cohort was generated (*N*_offspring included_ = 1,710,866), with 554,010 exposed to maternal overweight/obesity.

### Potential mediators

Mediators examined in this study were informed by previous research (as described in the introduction), including complications arising during pregnancy (pregnancy hypertensive diseases, placenta/membrane disorders, gestational infections); at the time of birth (labor complications, intrauterine hypoxia, small for gestational age [SGA], large for gestational age [LGA], preterm birth); and during the neonatal period (neonatal asphyxia comorbidities, neonatal hematological comorbidities, neonatal hypoglycemia, and neonatal infections). GDM was considered a mediator in the relationship between maternal overweight/obesity and offspring NDCs. A description of the ICD-9 and ICD-10 codes recorded in the MBR and NPR used to ascertain the mediators was described in Additional file 1: Table S1. An illustration of the rationale for potential causal pathways is depicted in Additional file 1: Figure S1. The Phi coefficients for the correlations between mediators can be found in Additional file 1: Figure S2.

### Potential confounders

We incorporated factors that might act as confounders in the associations between all exposure-outcome, exposure-mediator, and mediator-outcome relationships, based on findings from prior studies [7, 34, 43, 44]. These factors were children’s sex, birth year, maternal age, maternal birth country, birth order, household disposable income at birth, and maternal psychiatric history (Additional file 1: Table S1). Maternal BMI was considered as a confounder in the models associated with GDM.

### Statistical analysis

We compared the baseline differences of descriptive statistics (i.e., frequencies, percentages, means) between children who were exposed and unexposed to maternal PGDM, GDM, or adiposity and those who were not using *χ*^2^ test for proportions and analysis of variance for means.

We began by evaluating the potential for individual complications to explain the relationships between each metabolic condition (PGDM, GDM, and adiposity) and each NDC. Under assumptions of a counterfactual framework, we estimated the total effect (TE), natural indirect effects (NIE), and natural direct effects (NDE) of diabetes and elevated BMI on NDCs through individual mediators using a parametric regression approach (paramed package in Stata 16.0 [StataCorp]) accounting for confounders [45, 46]. The paramed package took interactions between exposures and mediators into account [47]. The magnitude of mediation was quantified by calculating log(NIE)/log(TE) for each mediator. We used bootstrapping with 500 replications to correct standard errors (SEs) for all direct and indirect effects. We applied the Bonferroni correction method to adjust the *P*-values [48]. With 12 mediators in consideration, the corrected *P*-value threshold becomes 0.004 (0.05/12).

To examine the joint effects of multiple mediators and accommodate cases in which multiple mediators affect one another, a weighting-based method proposed by VanderWeele and Vansteelandt [45, 49] was applied using the SAS 9.3 software (SAS Institute, Cary NC) (Additional file 1: Figure S3). SEs for all mediation effect estimates were estimated using 100 bootstrap iterations. We tested the change in proportions mediated in the associations between PGDM, GDM, adiposity, and NDCs by adding interaction terms of mediators and exposures in the models, which resulted in a < 1% change in proportions. We therefore did not include interaction terms in our final models for multiple mediation analyses. To ensure the robustness of our multiple mediation analysis findings considering a corrected *P*-value threshold of 0.004, we applied a 99.6% confidence interval (CI) and re-examined the joint mediating effects on associations between maternal metabolic conditions and NDCs when the 95% CI did not encompass the value of 1.

Several sensitivity analyses were performed. To assess the bias due to potential variation in disease coverage in ICD-9 (1987–1996) and ICD-10 (1997–2018) and an improved ascertainment of cases due to the introduction of the National Outpatient Register from 1997, we replicated our results among those born no earlier than 1997 when ICD-10 was introduced. As pregestational T1DM accounted for most of the PGDM cases (78.22%), we replicated our results using only T1DM as exposure. To address potential biases arising from siblings with non-independent NDC risks, we replicated our analysis with a sample of 785,500 children, each randomly selected from families with multiple siblings, and 514,192 children who were the only child in their families. This ensured that each family only contributed one child to the analysis and that assumptions of independence between the observations could be satisfied.

## Results

### Sample characteristics

In the full cohort of 2,352,969 offspring born to 1,299,692 mothers (Fig. 1), 135,832 (5.77%) were diagnosed with NDCs. Among those diagnosed, 45,654 (33.6%) received a diagnosis of autism, 20,764 (15.3%) with ID, and 101,986 (75.1%) with ADHD.

### The associations between maternal metabolic conditions and potential mediators

**Fig. 2:**
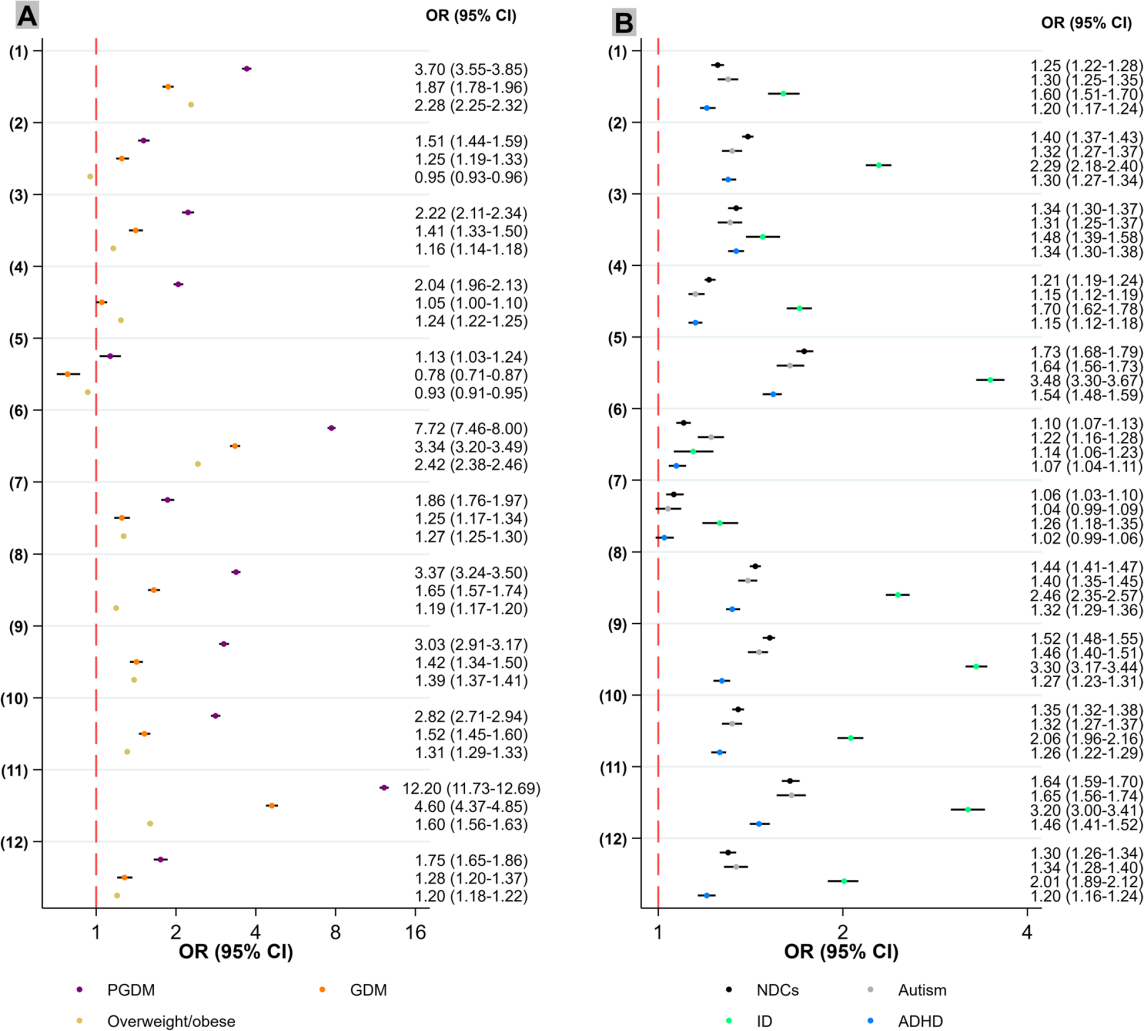
Visualization of associations between maternal metabolic conditions, offspring NDCs, and obstetric and neonatal complications. Logistic regression, with standard errors adjusted using a robust sandwich estimator. The *X*-axis is on a logarithmic scale. *Y*-axis labels represent the following: (1) pregnancy hypertensive diseases; (2) placenta/membrane disorders; (3) gestational infection; (4) intrauterine hypoxia; (5) SGA; (6) LGA; (7) labor complications; (8) preterm birth; (9) neonatal asphyxia comorbidities; (10) neonatal hematological comorbidities; (11) neonatal hypoglycemia; (12) neonatal infection. **A** A visualization of associations of maternal metabolic conditions as exposures with obstetric and neonatal complications as outcomes. Adjusted for the child’s sex, birthyear, maternal age, birth order, maternal birth country, and disposable income at birth. The associations related to GDM were further adjusted for maternal BMI. **B** A visualization of associations of obstetric and neonatal complications as exposures with offspring NDCs as outcomes. Adjusted for child’s sex, birthyear, maternal age, birth order, maternal birth country, disposable income at birth, and maternal psychiatric history

The prevalences of all covariates and incidence of potential mediators varied between exposed and unexposed groups (PGDM/GDM versus no-diabetes, and adiposity versus normal-weight), except for the child’s sex (Table 1). For example, LGA was more common among those exposed to maternal PGDM (19.9%) or GDM (12.5%) compared to those without maternal diabetes exposure (3.3%). LGA was also somewhat more common among children exposed to maternal overweight/obesity (6.0%) compared to those with normal-weight mothers (2.5%). Neonatal asphyxia conditions were more common among those exposed to maternal PGDM (11.4%) or GDM (6.3%) compared to those with no maternal diabetes exposure (4.1%). They were also more common among those exposed to high maternal adiposity (4.8%) compared to those with normal-weight mothers (3.7%). In adjusted models, all exposures were associated with increased odds of different mediators, except a marginally decreased odds of placenta/membrane disorders for adiposity and SGA for GDM and adiposity (Fig. 2A; Additional file 1: Table S2A). PGDM tended to be associated with the highest odds of each potentially mediating complication, followed by GDM and maternal overweight/obesity. For example, elevated odds of LGA were associated with PGDM (OR 7.72, 95% CI 7.46–8.00), followed by GDM (OR 3.34, 95% CI 3.20–3.49) and maternal overweight/obesity (2.42, 95% CI 2.38–2.46). The highest odds of neonatal asphyxia comorbidities were associated with PGDM (OR 3.03, 95% CI 2.91–3.17), followed by GDM (OR 1.42, 95% CI 1.34–1.50) and maternal overweight/obesity (1.39, 95% CI 1.37–1.41).

**Table 1:** Characteristics of the study sample over exposures

	**No diabetes**	**PGDM**	**GDM**	**Normal weight**	**Adiposity**
	**(*n*=2309663)**	**(*n*=22127)**	**(*n*=21179)**	**(*n*=1156856)**	**(*n*=554010)**
**Characteristics**					
**Any** **NDCs**	132502 (5.7%)	1814 (8.2%)	1516 (7.2%)	58241 (5.0%)	37690 (6.8%)
**Autism**	44469 (1.9%)	608 (2.7%)	577 (2.7%)	19765 (1.7%)	12777 (2.3%)
**ADHD**	99634 (4.3%)	1318 (6.0%)	1034 (4.9%)	44014 (3.8%)	28731 (5.2%)
**ID**	20092 (0.9%)	366 (1.7%)	306 (1.4%)	7952 (0.7%)	6029 (1.1%)
**Child’s** **sex**					
Male	1185538 (51.3%)	11307 (51.1%)	11026 (52.1%)	593437 (51.3%)	284691 (51.4%)
Female	1124125 (48.7%)	10820 (48.9%)	10153 (47.9%)	563419 (48.7%)	269319 (48.6%)
**Birthyear**					
1987-1992	647353 (28.0%)	6073 (27.4%)	5251 (24.8%)	228965 (19.8%)	56782 (10.2%)
1993-1998	553617 (24.0%)	5124 (23.2%)	5070 (23.9%)	312194 (27.0%)	138007 (24.9%)
1999-2004	515651 (22.3%)	5027 (22.7%)	4793 (22.6%)	281640 (24.3%)	159325 (28.8%)
2005-2010	593042 (25.7%)	5903 (26.7%)	6065 (28.6%)	334057 (28.9%)	199896 (36.1%)
**Maternal age, mean (SD)**	29.2 (5.1)	29.9 (5.4)	31.7 (5.4)	29.3 (5.0)	29.9 (5.2)
**Maternal birth country**					
Nordic	1992882 (86.3%)	18736 (84.7%)	15142 (71.5%)	1007793 (87.1%)	466840 (84.3%)
Europe	97710 (4.2%)	545 (2.5%)	1125 (5.3%)	50909 (4.4%)	22642 (4.1%)
Africa	41345 (1.8%)	815 (3.7%)	1047 (4.9%)	15989 (1.4%)	15099 (2.7%)
Asia	148144 (6.4%)	1803 (8.1%)	3432 (16.2%)	69126 (6.0%)	41116 (7.4%)
Other	29582 (1.3%)	228 (1.0%)	433 (2.0%)	13039 (1.1%)	8313 (1.5%)
**Disposable income at birth**					
1 (lowest)	322536 (14.0%)	3474 (15.7%)	4199 (19.8%)	137296 (11.9%)	79115 (14.3%)
2	473269 (20.5%)	5136 (23.2%)	5249 (24.8%)	221609 (19.2%)	132021 (23.8%)
3	496262 (21.5%)	4886 (22.1%)	4381 (20.7%)	246102 (21.3%)	133214 (24.0%)
4	507824 (22.0%)	4698 (21.2%)	3988 (18.8%)	265800 (23.0%)	121770 (22.0%)
5 (highest)	509772 (22.1%)	3933 (17.8%)	3362 (15.9%)	286049 (24.7%)	87890 (15.9%)
**Birth order**					
1	992824 (43.0%)	8530 (38.6%)	7187 (33.9%)	527609 (45.6%)	207246 (37.4%)
2	837502 (36.3%)	7782 (35.2%)	7083 (33.4%)	420561 (36.4%)	205141 (37.0%)
>=3	479337 (20.8%)	5815 (26.3%)	6909 (32.6%)	208686 (18.0%)	141623 (25.6%)
**Maternal psychiatric history**	115444 (5.0%)	1968 (8.9%)	1414 (6.7%)	56868 (4.9%)	35241 (6.4%)
**Mediators**					
**Pregnancy hypertensive diseases**	95092 (4.1%)	2930 (13.2%)	1860 (8.8%)	37923 (3.3%)	37311 (6.7%)
**Placenta/membrane disorders**	112498 (4.9%)	1596 (7.2%)	1341 (6.3%)	56768 (4.9%)	26342 (4.8%)
**Gestational infection**	82081 (3.6%)	1664 (7.5%)	1172 (5.5%)	41343 (3.6%)	24345 (4.4%)
**Intrauterine hypoxia**	163647 (7.1%)	2848 (12.9%)	1710 (8.1%)	82377 (7.1%)	45047 (8.1%)
**SGA**	53530 (2.3%)	485 (2.2%)	387 (1.8%)	25716 (2.2%)	10656 (1.9%)
**LGA**	75127 (3.3%)	4403 (19.9%)	2646 (12.5%)	28604 (2.5%)	33445 (6.0%)
**Labour complications**	77562 (3.4%)	1292 (5.8%)	889 (4.2%)	38006 (3.3%)	20748 (3.7%)
**Preterm birth**	108050 (4.7%)	3225 (14.6%)	1725 (8.1%)	49685 (4.3%)	26713 (4.8%)
**Neonatal asphyxia comorbidities**	95575 (4.1%)	2529 (11.4%)	1338 (6.3%)	43125 (3.7%)	26814 (4.8%)
**Neonatal hematological comorbidities**	107856 (4.7%)	2648 (12.0%)	1635 (7.7%)	50096 (4.3%)	30431 (5.5%)
**Neonatal hypoglycemia**	37130 (1.6%)	3530 (16.0%)	1754 (8.3%)	18457 (1.6%)	14953 (2.7%)
**Neonatal infection**	76135 (3.3%)	1252 (5.7%)	952 (4.5%)	37333 (3.2%)	22206 (4.0%)

The chi-squared test was used to test for differences in the proportions of each categorical covariate, first across categories of maternal diabetes (no diabetes, PGDM, and GDM) and then across maternal BMI categories (normal weight compared to overweight or obese). *P*-values were < 0.001 for all covariates, except for the child’s sex, where *P* = 0.083 (for maternal diabetes) and *P* = 0.27 (for maternal adiposity)

### The associations between potential mediators and offspring NDCs

The prevalences of all covariates and potential mediators also varied between children affected by NDCs compared to those who were not (Additional file 1: Table S3). For example, the proportion of children who were SGA was higher among those affected by autism (3.7%), ADHD (3.3%), and ID (7.4%) compared to children without any NDCs (2.2%). The proportion of children who had experienced preterm birth was higher among those affected by autism (6.7%), ADHD (6.3%), and ID (10.8%) compared to children without any NDCs (4.7%). Similarly, the proportion of children who experienced neonatal asphyxia comorbidities was also higher among those affected by autism (6.4%), ADHD (5.4%), and ID (12.6%), compared to children without any NDCs (4.1%). In adjusted models (Fig. 2B; Additional file 1: Table S2B), all potentially mediating complications were associated with increased odds of NDCs, except labor complications for autism and ADHD. For example, SGA was associated with increased odds of autism (OR 1.64, 95% CI 1.56–1.73), ADHD (OR 1.54 95% CI 1.48–1.59), and ID (OR 3.48, 95% CI 3.30–3.67). Preterm birth was associated with increased odds of autism (OR 1.40, 95% CI 1.35–1.45), ADHD (OR 1.32, 95% CI 1.29–1.36), and ID (OR 2.46, 95% CI 2.35–2.57). Neonatal asphyxia comorbidities were associated with increased odds of autism (OR 1.46, 95% CI 1.40–1.51), ADHD (OR 1.27, 95% CI 1.23–1.31), and ID (OR 3.30, 95% CI 3.17–3.44) (Fig. 2B; Additional file 1: Table S2B).

**Fig. 3:**
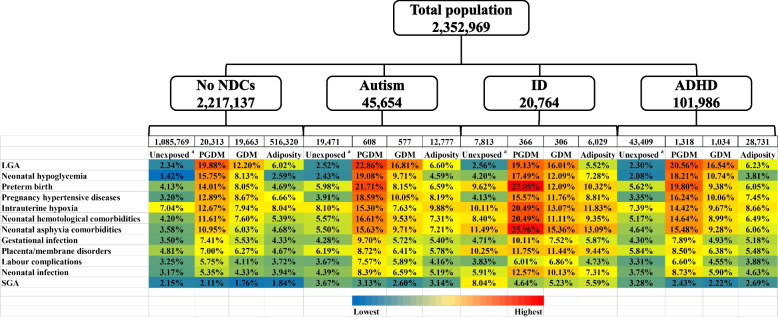
Percentages (%) of mediators by exposure group for each outcome in the full cohort. Superscript lowercase letter a “^a^” indicates the following: the unexposed group comprised offspring whose mothers were not exposed to PGDM, GDM, or adiposity

After stratifying the population according to children’s NDC outcomes, we depict the incidence of potentially mediating complications among unexposed (i.e., those with normal maternal BMI and without exposure to PGDM or GDM) and the groups exposed to PGDM, GDM, or high maternal adiposity (Fig. 3). Compared to the unexposed group, the proportion of mediators was generally higher in the exposed groups regardless of outcome, except SGA. Moreover, the incidence of the mediators tended to be higher among children affected by NDCs compared to unaffected children, across all categories including those exposed to maternal PGDM, GDM, or high adiposity. For example, considering those children exposed to maternal PGDM, the proportion of those who experienced neonatal asphyxia tended to be higher among those later diagnosed with autism (15.63%), ADHD (15.48%), or ID (25.96%), in contrast to those unaffected by NDCs (10.95%).

### Mediating effects of single mediators

**Fig. 4:**
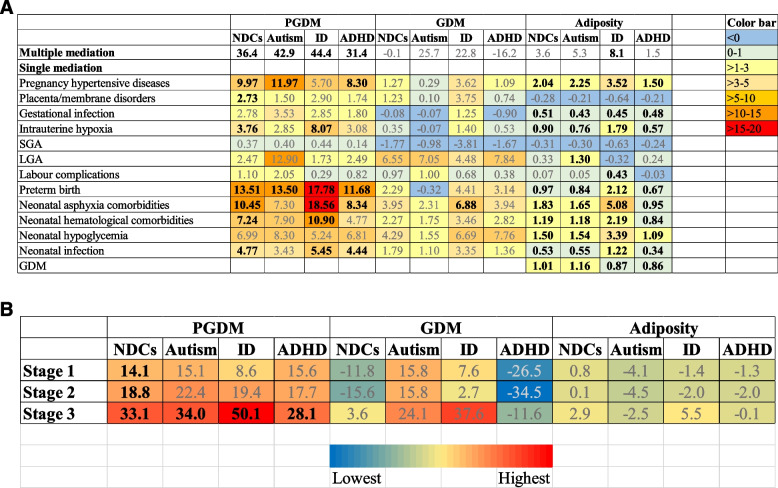
Visualization and summary of proportions mediated in single and multiple mediation analyses. **A** A summary and visualization of proportions mediated in the single and multiple mediation analyses. Adjusted for child’s sex, birthyear, maternal age, birth order, maternal birth country, disposable income at birth, and maternal psychiatric history. Analyses for GDM were additionally adjusted for maternal BMI. Multiple mediators included in the analysis were subsequently presented as individual mediators in the subsequent single mediation analyses for each exposure and outcome. Proportions mediated were presented in bold for NIEs with a 99.6% CI not encompassing a value of 1 in multiple mediation analysis (see Additional file 1: Table S4) and for *P*_Bonferroni corrected_ < 0.004 in single mediation analysis, with both NDE and NIE having the same direction. **B** A summary and visualization of multiple mediation analyses of mediators at different stages. Adjusted for child’s sex, birthyear, maternal age, birth order, maternal birth country, disposable income at birth, and maternal psychiatric history. Analyses for GDM were additionally adjusted for maternal BMI. The rationale for including different mediators in each multiple mediation analysis was described in Additional file 1: Figure S1. Mediators in stage 1 for PGDM and GDM included the following: pregnancy hypertensive diseases, placenta/membrane disorders, and gestational infection. Mediators in stage 1 for adiposity include: pregnancy hypertensive diseases, placenta/membrane disorders, gestational infection, and GDM. Mediators in stage 2 included the following: SGA, and LGA, intrauterine hypoxia, labor complications, and preterm birth. Mediators in stage 3 included the following: neonatal asphyxia comorbidities, neonatal hematological comorbidities, neonatal hypoglycemia, and neonatal infection. Proportions mediated were presented in bold for NIEs with a 99.6% CI not encompassing a value of 1 in multiple mediation analysis (see Additional file 1: Table S4)

The mediating effect of each complication was presented in Table 2 and Fig. 4A, based on the mediation estimates of the indirect and total associations calculated for each relationship. As described in Fig. 2A, PGDM showed stronger associations with obstetric complications (including pregnancy hypertensive diseases, placenta/membrane disorders, and gestational infections) when compared to GDM and adiposity. This association was particularly evident for pregnancy hypertensive diseases. According to Table 2 and Fig. 4A, pregnancy hypertensive diseases had a greater mediating effect compared to placenta/membrane disorders and gestational infection in the associations between maternal metabolic conditions and NDCs. For example, the proportions mediated by pregnancy hypertensive diseases were highest for the relationships between PGDM and the NDCs, ranging from 5.70% for ID to 11.97% for autism, and were lower for the relationships between high maternal adiposity and children’s NDC outcomes, ranging from 1.50% for ADHD to 3.52% for ID. After applying the Bonferroni correction for *P* values (*P* < 0.004), none of the NIEs related to GDM were statistically significant.

**Table 2:** Mediation analysis of the associations between maternal metabolic conditions and offspring NDCs: single- and multiple-component approaches

	**Any NDCs**		**Autism**		**ID**		**ADHD**	
**Multiple mediators**^**a**^	**OR (95% CI)**	***P*-value**	**OR (95% CI)**	***P*-value**	**OR (95% CI)**	***P*-value**	**OR (95% CI)**	***P*-value**
**A PGDM**								
NDE	1.26 (1.26–1.27)	NA	1.23 (1.22–1.25)	NA	1.38 (1.36–1.40)	NA	1.27 (1.26–1.28)	NA
NIE	1.14 (1.09–1.20)	NA	1.17 (1.07–1.26)	NA	1.29 (1.18–1.41)	NA	1.12 (1.04–1.18)	NA
TE	1.44 (1.38–1.51)	NA	1.44 (1.31–1.55)	NA	1.77 (1.63–1.96)	NA	1.42 (1.32–1.50)	NA
Proportion mediated, %	36.4**		42.9**		44.4**		31.4**	
**Single mediators**^**b**^								
**Pregnancy hypertensive diseases**								
NDE	1.38 (1.31–1.45)	< 0.001	1.36 (1.25–1.49)	< 0.001	1.76 (1.55–1.96)	< 0.001	1.35 (1.27–1.43)	< 0.001
NIE	1.04 (1.02–1.05)	< 0.001	1.04 (1.02–1.07)	0.001	1.03 (1.00–1.07)	0.038	1.03 (1.01–1.05)	0.001
TE	1.43 (1.36–1.51)	< 0.001	1.41 (1.31–1.55)	< 0.001	1.83 (1.61–2.03)	< 0.001	1.39 (1.31–1.47)	< 0.001
Proportion mediated, %	9.97**		11.97**		5.70*		8.30**	
**Placenta/membrane disorders**								
NDE	1.42 (1.35–1.49)	< 0.001	1.41 (1.31–1.53)	< 0.001	1.79 (1.59–2.01)	< 0.001	1.38 (1.31–1.46)	< 0.001
NIE	1.01 (1.01–1.02)	< 0.001	1.01 (1.00–1.01)	0.155	1.02 (1.01–1.03)	0.004	1.01 (1.00–1.01)	0.034
TE	1.43 (1.36–1.51)	< 0.001	1.42 (1.32–1.55)	< 0.001	1.83 (1.61–2.05)	< 0.001	1.39 (1.32–1.47)	< 0.001
Proportion mediated, %	2.73**		1.50		2.90*		1.74*	
**Gestational infection**								
NDE	1.42 (1.35–1.49)	< 0.001	1.40 (1.30–1.51)	< 0.001	1.79 (1.57–1.98)	< 0.001	1.39 (1.31–1.46)	< 0.001
NIE	1.01 (1.00–1.02)	0.008	1.01 (1.00–1.03)	0.047	1.02 (1.00–1.04)	0.044	1.01 (1.00–1.01)	0.143
TE	1.43 (1.36–1.51)	< 0.001	1.42 (1.31–1.54)	< 0.001	1.82 (1.61–2.02)	< 0.001	1.39 (1.32–1.47)	< 0.001
Proportion mediated, %^b^	2.78*		3.53*		2.85*		1.80	
**Intrauterine hypoxia**								
NDE	1.41 (1.34–1.49)	< 0.001	1.40 (1.29–1.54)	< 0.001	1.74 (1.54–1.93)	< 0.001	1.38 (1.30–1.46)	< 0.001
NIE	1.01 (1.01–1.02)	0.003	1.01 (1.00–1.03)	0.163	1.05 (1.02–1.07)	< 0.001	1.01 (1.00–1.02)	0.048
TE	1.43 (1.36–1.51)	< 0.001	1.42 (1.31–1.55)	< 0.001	1.82 (1.61–2.03)	< 0.001	1.39 (1.31–1.47)	< 0.001
Proportion mediated, %	3.76**		2.85		8.07**		3.08*	
**SGA**								
NDE	1.42 (1.34–1.51)	< 0.001	1.35 (1.22–1.46)	< 0.001	1.82 (1.58–2.01)	< 0.001	1.38 (1.29–1.48)	< 0.001
NIE	1.00 (1.00–1.00)	0.104	1.00 (1.00–1.01)	0.236	1.00 (1.00–1.01)	0.138	1.00 (1.00–1.00)	0.399
TE	1.42 (1.35–1.51)	< 0.001	1.35 (1.22–1.46)	< 0.001	1.83 (1.58–2.01)	< 0.001	1.38 (1.29–1.48)	< 0.001
Proportion mediated, %	0.37		0.40		0.44		0.14	
**LGA**								
NDE	1.42 (1.35–1.50)	< 0.001	1.36 (1.22–1.48)	< 0.001	1.87 (1.65–2.09)	< 0.001	1.39 (1.30–1.47)	< 0.001
NIE	1.01 (0.99–1.03)	0.398	1.05 (1.01–1.09)	0.022	1.01 (0.97–1.06)	0.636	1.01 (0.99–1.04)	0.489
TE	1.44 (1.37–1.51)	< 0.001	1.42 (1.30–1.53)	< 0.001	1.89 (1.68–2.10)	< 0.001	1.40 (1.32–1.49)	< 0.001
Proportion mediated, %	2.47		12.90*		1.73		2.49	
**Labour complications**								
NDE	1.43 (1.35–1.50)	< 0.001	1.41 (1.30–1.53)	< 0.001	1.82 (1.60–2.03)	< 0.001	1.39 (1.31–1.47)	< 0.001
NIE	1.00 (1.00–1.01)	0.163	1.01 (1.00–1.02)	0.144	1.00 (0.99–1.01)	0.764	1.00 (1.00–1.01)	0.389
TE	1.43 (1.36–1.51)	< 0.001	1.42 (1.32–1.55)	< 0.001	1.82 (1.61–2.03)	< 0.001	1.39 (1.31–1.47)	< 0.001
Proportion mediated, %	1.10		2.05		0.29		0.82	
**Preterm birth**								
NDE	1.37 (1.30–1.44)	< 0.001	1.38 (1.27–1.51)	< 0.001	1.66 (1.49–1.88)	< 0.001	1.34 (1.26–1.42)	< 0.001
NIE	1.05 (1.03–1.07)	< 0.001	1.05 (1.03–1.08)	< 0.001	1.12 (1.07–1.17)	< 0.001	1.04 (1.02–1.06)	< 0.001
TE	1.44 (1.37–1.52)	< 0.001	1.45 (1.33–1.58)	< 0.001	1.86 (1.68–2.08)	< 0.001	1.40 (1.31–1.47)	< 0.001
Proportion mediated, %	13.51**		13.50**		17.78**		11.68**	
**Neonatal asphyxia comorbidities**								
NDE	1.38 (1.31–1.45)	< 0.001	1.38 (1.27–1.52)	< 0.001	1.63 (1.43–1.85)	< 0.001	1.35 (1.27–1.43)	< 0.001
NIE	1.04 (1.02–1.05)	< 0.001	1.03 (1.00–1.05)	0.013	1.12 (1.08–1.16)	< 0.001	1.03 (1.01–1.05)	< 0.001
TE	1.43 (1.36–1.51)	< 0.001	1.42 (1.31–1.54)	< 0.001	1.82 (1.61–2.03)	< 0.001	1.39 (1.31–1.47)	< 0.001
Proportion mediated, %	10.45**		7.30*		18.56**		8.34**	
**Neonatal hematological comorbidities**								
NDE	1.39 (1.32–1.47)	< 0.001	1.38 (1.27–1.50)	< 0.001	1.71 (1.52–1.92)	< 0.001	1.37 (1.28–1.44)	< 0.001
NIE	1.03 (1.01–1.04)	< 0.001	1.03 (1.01–1.05)	0.008	1.07 (1.03–1.10)	< 0.001	1.02 (1.00–1.03)	0.021
TE	1.43 (1.36–1.51)	< 0.001	1.41 (1.31–1.54)	< 0.001	1.82 (1.61–2.03)	< 0.001	1.39 (1.31–1.47)	< 0.001
Proportion mediated, %	7.24**		7.90*		10.90**		4.77*	
**Neonatal hypoglycemia**								
NDE	1.39 (1.32–1.47)	< 0.001	1.37 (1.26–1.52)	< 0.001	1.77 (1.57–2.01)	< 0.001	1.36 (1.27–1.44)	< 0.001
NIE	1.03 (1.01–1.05)	0.008	1.03 (1.00–1.06)	0.064	1.03 (1.00–1.08)	0.129	1.02 (1.00–1.05)	0.036
TE	1.43 (1.36–1.50)	< 0.001	1.41 (1.31–1.55)	< 0.001	1.83 (1.61–2.03)	< 0.001	1.39 (1.31–1.47)	< 0.001
Proportion mediated, %	6.99*		8.30		5.24		6.81*	
**Neonatal infection**								
NDE	1.41 (1.34–1.48)	< 0.001	1.40 (1.30–1.53)	< 0.001	1.77 (1.57–1.97)	< 0.001	1.37 (1.29–1.45)	< 0.001
NIE	1.02 (1.01–1.03)	< 0.001	1.01 (1.00–1.02)	0.012	1.03 (1.02–1.05)	< 0.001	1.01 (1.01–1.02)	< 0.001
TE	1.43 (1.36–1.51)	< 0.001	1.42 (1.32–1.55)	< 0.001	1.83 (1.61–2.04)	< 0.001	1.39 (1.32–1.47)	< 0.001
Proportion mediated, %	4.77**		3.43*		5.45**		4.44**	
**B GDM**								
NDE	1.19 (1.18–1.19)	NA	1.25 (1.24–1.26)	NA	1.26 (1.25–1.28)	NA	1.14 (1.13–1.14)	NA
NIE	1.00 (0.93–1.07)	NA	1.08 (0.98–1.19)	NA	1.07 (0.96–1.26)	NA	0.98 (0.90–1.10)	NA
TE	1.19 (1.10–1.27)	NA	1.35 (1.22–1.48)	NA	1.35 (1.21–1.60)	NA	1.12 (1.03–1.25)	NA
Proportion mediated, %	-0.1		25.7		22.8		-16.2	
**Single mediation**^**b**^								
**Pregnancy hypertensive diseases**								
NDE	1.23 (1.16–1.28)	< 0.001	1.31 (1.19–1.42)	< 0.001	1.34 (1.20–1.51)	< 0.001	1.16 (1.08–1.24)	< 0.001
NIE	1.00 (1.00–1.01)	0.345	1.00 (0.99–1.01)	0.845	1.01 (1.00–1.03)	0.110	1.00 (1.00–1.01)	0.604
TE	1.23 (1.16–1.29)	< 0.001	1.32 (1.19–1.42)	< 0.001	1.36 (1.22–1.53)	< 0.001	1.17 (1.09–1.24)	< 0.001
Proportion mediated, %	1.27		0.29		3.62		1.09	
**Placenta/membrane disorders**								
NDE	1.23 (1.15–1.28)	< 0.001	1.31 (1.18–1.41)	< 0.001	1.35 (1.20–1.51)	< 0.001	1.16 (1.08–1.24)	< 0.001
NIE	1.00 (1.00–1.01)	0.074	1.00 (1.00–1.00)	0.883	1.01 (1.00–1.02)	0.005	1.00 (1.00–1.00)	0.471
TE	1.23 (1.16–1.28)	< 0.001	1.31 (1.18–1.41)	< 0.001	1.37 (1.22–1.53)	< 0.001	1.17 (1.08–1.24)	< 0.001
Proportion mediated, %	1.23		0.10		3.75*		0.74	
**Maternal infection**								
NDE	1.23 (1.26–1.29)	< 0.001	1.32 (1.18–1.42)	< 0.001	1.36 (1.21–1.52)	< 0.001	1.17 (1.09–1.25)	< 0.001
NIE	1.00 (1.00–1.00)	0.901	1.00 (1.00–1.00)	0.929	1.00 (1.00–1.01)	0.254	1.00 (1.00–1.00)	0.343
TE	1.23 (1.15–1.29)	< 0.001	1.32 (1.18–1.42)	< 0.001	1.36 (1.22–1.52)	< 0.001	1.17 (1.09–1.24)	< 0.001
Proportion mediated, %	-0.08		-0.07		1.25		-0.90	
**Intrauterine hypoxia**								
NDE	1.23 (1.15–1.28)	< 0.001	1.32 (1.19–1.42)	< 0.001	1.35 (1.21–1.52)	< 0.001	1.16 (1.08–1.24)	< 0.001
NIE	1.00 (1.00–1.00)	0.157	1.00 (1.00–1.00)	0.523	1.00 (1.00–1.01)	0.101	1.00 (1.00–1.00)	0.173
TE	1.23 (1.15–1.28)	< 0.001	1.32 (1.19–1.42)	< 0.001	1.36 (1.21–1.52)	< 0.001	1.16 (1.08–1.24)	< 0.001
Proportion mediated, %	0.35		-0.07		1.40		0.53	
**SGA**								
NDE	1.20 (1.13–1.27)	< 0.001	1.27 (1.16–1.39)	< 0.001	1.35 (1.19–1.51)	< 0.001	1.14 (1.06–1.22)	< 0.001
NIE	1.00 (0.99–1.00)	0.029	1.00 (0.99–1.00)	0.215	0.99 (0.98–1.00)	0.014	1.00 (0.99–1.00)	0.164
TE	1.20 (1.13–1.27)	< 0.001	1.27 (1.15–1.38)	< 0.001	1.33 (1.17–1.50)	< 0.001	1.14 (1.06–1.22)	< 0.001
Proportion mediated, %	-1.77		-0.98		-3.81		-1.67	
**LGA**								
NDE	1.21 (1.15–1.27)	< 0.001	1.29 (1.17–1.42)	< 0.001	1.35 (1.18–1.51)	< 0.001	1.15 (1.08–1.23)	< 0.001
NIE	1.01 (1.00–1.02)	0.011	1.02 (1.00–1.04)	0.027	1.01 (0.99–1.04)	0.209	1.01 (1.00–1.02)	0.052
TE	1.23 (1.17–1.29)	< 0.001	1.31 (1.20–1.45)	< 0.001	1.37 (1.20–1.53)	< 0.001	1.16 (1.10–1.25)	< 0.001
Proportion mediated, %	6.55*		7.05*		4.48		7.84	
**Labour complications**								
NDE	1.23 (1.15–1.28)	< 0.001	1.31 (1.17–1.40)	< 0.001	1.36 (1.21–1.52)	< 0.001	1.16 (1.08–1.24)	< 0.001
NIE	1.00 (1.00–1.00)	0.085	1.00 (1.00–1.01)	0.134	1.00 (1.00–1.01)	0.151	1.00 (1.00–1.00)	0.609
TE	1.23 (1.15–1.28)	< 0.001	1.31 (1.18–1.40)	< 0.001	1.36 (1.21–1.52)	< 0.001	1.17 (1.09–1.24)	< 0.001
Proportion mediated, %	0.97		1.00		0.68		0.38	
**Preterm birth**								
NDE	1.23 (1.16–1.30)	< 0.001	1.33 (1.20–1.45)	< 0.001	1.39 (1.24–1.56)	< 0.001	1.16 (1.08–1.23)	< 0.001
NIE	1.00 (1.00–1.01)	0.120	1.00 (0.99–1.01)	0.833	1.02 (1.00–1.03)	0.045	1.00 (1.00–1.01)	0.204
TE	1.23 (1.16–1.30)	< 0.001	1.33 (1.20–1.45)	< 0.001	1.41 (1.26–1.59)	< 0.001	1.16 (1.09–1.24)	< 0.001
Proportion mediated, %	2.29		-0.32		4.41*		3.14	
**Neonatal asphyxia comorbidities**								
NDE	1.22 (1.15–1.28)	< 0.001	1.30 (1.18–1.41)	< 0.001	1.33 (1.19–1.50)	< 0.001	1.15 (1.07–1.23)	< 0.001
NIE	1.01 (1.00–1.01)	< 0.001	1.01 (1.00–1.01)	0.035	1.02 (1.01–1.03)	< 0.001	1.01 (1.00–1.01)	0.012
TE	1.23 (1.15–1.28)	< 0.001	1.31 (1.18–1.41)	< 0.001	1.36 (1.22–1.53)	< 0.001	1.16 (1.08–1.24)	< 0.001
Proportion mediated, %	3.95**		2.31*		6.88**		3.94*	
**Neonatal hematological comorbidities**								
NDE	1.22 (1.15–1.28)	< 0.001	1.31 (1.18–1.41)	< 0.001	1.35 (1.20–1.52)	< 0.001	1.16 (1.08–1.24)	< 0.001
NIE	1.00 (1.00–1.01)	0.053	1.00 (1.00–1.01)	0.201	1.01 (1.00–1.02)	0.059	1.00 (1.00–1.01)	0.136
TE	1.23 (1.16–1.28)	< 0.001	1.31 (1.18–1.41)	< 0.001	1.37 (1.22–1.53)	< 0.001	1.17 (1.08–1.24)	< 0.001
Proportion mediated, %	2.27		1.75		3.46		2.82	
**Neonatal hypoglycemia**								
NDE	1.22 (1.15–1.28)	< 0.001	1.31 (1.18–1.41)	< 0.001	1.34 (1.19–1.50)	< 0.001	1.15 (1.06–1.23)	< 0.001
NIE	1.01 (1.00–1.02)	0.055	1.00 (0.99–1.02)	0.523	1.02 (1.00–1.05)	0.057	1.01 (1.00–1.02)	0.042
TE	1.23 (1.15–1.28)	< 0.001	1.31 (1.18–1.41)	< 0.001	1.36 (1.22–1.53)	< 0.001	1.16 (1.08–1.24)	< 0.001
Proportion mediated, %	4.29		1.55		6.69		7.76*	
**Neonatal infection**								
NDE	1.22 (1.15–1.28)	< 0.001	1.31 (1.18–1.40)	< 0.001	1.34 (1.20–1.50)	< 0.001	1.16 (1.08–1.24)	< 0.001
NIE	1.00 (1.00–1.01)	0.010	1.00 (1.00–1.01)	0.117	1.01 (1.00–1.02)	0.006	1.00 (1.00–1.01)	0.143
TE	1.23 (1.15–1.28)	< 0.001	1.31 (1.18–1.40)	< 0.001	1.36 (1.21–1.52)	< 0.001	1.16 (1.08–1.24)	< 0.001
Proportion mediated, %	1.79*		1.10		3.35*		1.36	
**C Adiposity**								
NDE	1.34 (1.33–1.36)	NA	1.34 (1.32–1.36)	NA	1.52 (1.49–1.56)	NA	1.36 (1.34–1.37)	NA
NIE	1.01 (1.00–1.02)	NA	1.02 (1.00–1.03)	NA	1.04 (1.01–1.07)	NA	1.00 (0.99–1.02)	NA
TE	1.36 (1.34–1.38)	NA	1.36 (1.33–1.39)	NA	1.58 (1.53–1.62)	NA	1.37 (1.34–1.39)	NA
Proportion mediated, %	3.6*		5.3*		8.1**		1.5	
**Single mediation**^**b**^								
**Pregnancy hypertensive diseases**								
NDE	1.36 (1.34–1.38)	< 0.001	1.36 (1.33–1.40)	< 0.001	1.56 (1.50–1.61)	< 0.001	1.37 (1.35–1.40)	< 0.001
NIE	1.01 (1.00–1.01)	< 0.001	1.01 (1.00–1.01)	< 0.001	1.02 (1.01–1.02)	< 0.001	1.00 (1.00–1.01)	< 0.001
TE	1.37 (1.35–1.39)	< 0.001	1.37 (1.34–1.41)	< 0.001	1.58 (1.53–1.64)	< 0.001	1.38 (1.36–1.40)	< 0.001
Proportion mediated, %	2.04**		2.25**		3.52**		1.50**	
**Placenta/membrane disorders**								
NDE	1.37 (1.35–1.39)	< 0.001	1.37 (1.34–1.41)	< 0.001	1.59 (1.54–1.65)	< 0.001	1.38 (1.36–1.40)	< 0.001
NIE	1.00 (1.00–1.00)	< 0.001	1.00 (1.00–1.00)	< 0.001	1.00 (1.00–1.00)	< 0.001	1.00 (1.00–1.00)	< 0.001
TE	1.37 (1.35–1.39)	< 0.001	1.37 (1.34–1.41)	< 0.001	1.58 (1.53–1.64)	< 0.001	1.38 (1.36–1.40)	< 0.001
Proportion mediated, %	-0.28		-0.21		-0.64		-0.21	
**Gestational infection**								
NDE	1.37 (1.35–1.39)	< 0.001	1.37 (1.34–1.41)	< 0.001	1.58 (1.53–1.64)	< 0.001	1.38 (1.36–1.40)	< 0.001
NIE	1.00 (1.00–1.00)	< 0.001	1.00 (1.00–1.00)	< 0.001	1.00 (1.00–1.00)	< 0.001	1.00 (1.00–1.00)	< 0.001
TE	1.37 (1.35–1.39)	< 0.001	1.37 (1.34–1.41)	< 0.001	1.58 (1.53–1.64)	< 0.001	1.38 (1.36–1.40)	< 0.001
Proportion mediated, %	0.51**		0.43**		0.45**		0.48**	
**Intrauterine hypoxia**								
NDE	1.37 (1.35–1.39)	< 0.001	1.37 (1.34–1.40)	< 0.001	1.57 (1.52–1.63)	< 0.001	1.38 (1.36–1.40)	< 0.001
NIE	1.00 (1.00–1.00)	< 0.001	1.00 (1.00–1.00)	< 0.001	1.01 (1.01–1.01)	< 0.001	1.00 (1.00–1.00)	< 0.001
TE	1.37 (1.35–1.39)	< 0.001	1.37 (1.34–1.41)	< 0.001	1.58 (1.53–1.64)	< 0.001	1.38 (1.36–1.40)	< 0.001
Proportion mediated, %	0.90**		0.76**		1.79**		0.57**	
**SGA**								
NDE	1.37 (1.35–1.39)	< 0.001	1.37 (1.33–1.39)	< 0.001	1.61 (1.55–1.66)	< 0.001	1.38 (1.36–1.40)	< 0.001
NIE	1.00 (1.00–1.00)	< 0.001	1.00 (1.00–1.00)	< 0.001	1.00 (1.00–1.00)	< 0.001	1.00 (1.00–1.00)	< 0.001
TE	1.37 (1.35–1.39)	< 0.001	1.36 (1.33–1.39)	< 0.001	1.60 (1.55–1.66)	< 0.001	1.38 (1.36–1.40)	< 0.001
Proportion mediated, %	-0.31		-0.30		-0.63		-0.24	
**LGA**								
NDE	1.38 (1.36–1.40)	< 0.001	1.37 (1.34–1.40)	< 0.001	1.62 (1.56–1.68)	< 0.001	1.38 (1.36–1.40)	< 0.001
NIE	1.00 (1.00–1.00)	0.146	1.00 (1.00–1.01)	0.001	1.00 (0.99–1.00)	0.365	1.00 (1.00–1.00)	0.332
TE	1.38 (1.36–1.40)	< 0.001	1.37 (1.35–1.41)	< 0.001	1.62 (1.56–1.68)	< 0.001	1.38 (1.36–1.40)	< 0.001
Proportion mediated, %	0.33		1.30**		-0.32		0.24	
**Labour complications**								
NDE	1.37 (1.35–1.39)	< 0.001	1.37 (1.34–1.41)	< 0.001	1.58 (1.53–1.64)	< 0.001	1.38 (1.36–1.40)	< 0.001
NIE	1.00 (1.00–1.00)	0.316	1.00 (1.00–1.00)	0.673	1.00 (1.00–1.00)	0.001	1.00 (1.00–1.00)	0.712
TE	1.37 (1.35–1.39)	< 0.001	1.37 (1.34–1.41)	< 0.001	1.58 (1.53–1.64)	< 0.001	1.38 (1.36–1.40)	< 0.001
Proportion mediated, %	0.07		0.05		0.43**		-0.03	
**Preterm birth**								
NDE	1.37 (1.35–1.39)	< 0.001	1.37 (1.33–1.40)	< 0.001	1.57 (1.51–1.62)	< 0.001	1.38 (1.36–1.41)	< 0.001
NIE	1.00 (1.00–1.00)	< 0.001	1.00 (1.00–1.00)	< 0.001	1.01 (1.01–1.01)	< 0.001	1.00 (1.00–1.00)	< 0.001
TE	1.37 (1.35–1.40)	< 0.001	1.37 (1.34–1.41)	< 0.001	1.59 (1.53–1.64)	< 0.001	1.38 (1.36–1.41)	< 0.001
Proportion mediated, %	0.97**		0.84**		2.12**		0.67**	
**Neonatal asphyxia comorbidities**								
NDE	1.37 (1.35–1.38)	< 0.001	1.36 (1.33–1.40)	< 0.001	1.55 (1.50–1.61)	< 0.001	1.38 (1.36–1.40)	< 0.001
NIE	1.01 (1.01–1.01)	< 0.001	1.01 (1.00–1.01)	< 0.001	1.02 (1.02–1.03)	< 0.001	1.00 (1.00–1.00)	< 0.001
TE	1.37 (1.35–1.39)	< 0.001	1.37 (1.34–1.41)	< 0.001	1.59 (1.53–1.64)	< 0.001	1.38 (1.36–1.40)	< 0.001
Proportion mediated, %	1.83**		1.65**		5.08**		0.95**	
**Neonatal hematological comorbidities**								
NDE	1.37 (1.35–1.39)	< 0.001	1.37 (1.34–1.40)	< 0.001	1.57 (1.52–1.63)	< 0.001	1.38 (1.36–1.40)	< 0.001
NIE	1.00 (1.00–1.00)	< 0.001	1.00 (1.00–1.00)	< 0.001	1.01 (1.01–1.01)	< 0.001	1.00 (1.00–1.00)	< 0.001
TE	1.37 (1.35–1.39)	< 0.001	1.37 (1.34–1.41)	< 0.001	1.59 (1.53–1.64)	< 0.001	1.38 (1.36–1.40)	< 0.001
Proportion mediated, %	1.19**		1.18**		2.19**		0.84**	
**Neonatal hypoglycemia**								
NDE	1.37 (1.35–1.38)	< 0.001	1.36 (1.33–1.40)	< 0.001	1.56 (1.50–1.61)	< 0.001	1.38 (1.36–1.40)	< 0.001
NIE	1.00 (1.00–1.01)	< 0.001	1.00 (1.00–1.01)	< 0.001	1.02 (1.01–1.02)	< 0.001	1.00 (1.00–1.00)	< 0.001
TE	1.37 (1.35–1.39)	< 0.001	1.37 (1.34–1.41)	< 0.001	1.58 (1.53–1.64)	< 0.001	1.38 (1.36–1.40)	< 0.001
Proportion mediated, %	1.50**		1.54**		3.39**		1.09**	
**Neonatal infection**								
NDE	1.37 (1.35–1.39)	< 0.001	1.37 (1.34–1.41)	< 0.001	1.58 (1.52–1.64)	< 0.001	1.38 (1.36–1.40)	< 0.001
NIE	1.00 (1.00–1.00)	< 0.001	1.00 (1.00–1.00)	< 0.001	1.01 (1.00–1.01)	< 0.001	1.00 (1.00–1.00)	< 0.001
TE	1.37 (1.35–1.39)	< 0.001	1.37 (1.34–1.41)	< 0.001	1.58 (1.53–1.64)	< 0.001	1.38 (1.36–1.40)	< 0.001
Proportion mediated, %	0.53**		0.55**		1.22**		0.34**	
**GDM**								
NDE	1.36 (1.34–1.38)	< 0.001	1.36 (1.33–1.40)	< 0.001	1.57 (1.52–1.62)	< 0.001	1.37 (1.35–1.40)	< 0.001
NIE	1.00 (1.00–1.00)	< 0.001	1.00 (1.00–1.01)	< 0.001	1.00 (1.00–1.01)	< 0.001	1.00 (1.00–1.00)	< 0.001
TE	1.37 (1.35–1.39)	< 0.001	1.37 (1.34–1.40)	< 0.001	1.57 (1.52–1.63)	< 0.001	1.38 (1.36–1.40)	< 0.001
Proportion mediated, %	1.01**		1.16**		0.87**		0.86**	

Adjusted for child’s sex, birthyear, maternal age, birth order, maternal birth country, disposable income at birth, and maternal psychiatric history. Analyses for GDM were additionally adjusted for maternal BMI

^a^Multiple mediators included in the analysis were subsequently presented as individual mediators in the subsequent single mediation analyses for each exposure and outcome. Mediated proportions were marked with “*” when the 95% CI for NIEs did not encompass a value of 1, and by “**” when the 99.6% CI for NIEs did not encompass a value of 1 (refer to Additional file 1: Table S4), with both NDE and NIE having the same direction

^b^Single mediation analysis. Proportions mediated were marked with “*” when P < 0.05 for NIE and with “**” when P_Bonferroni corrected_ < 0.004 for NIE, with both NDE and NIE having the same direction

PGDM also showed a stronger association with complications around the birth period compared to GDM and adiposity, particularly with regard to LGA and preterm birth (Fig. 2A; Additional file 1: Table S2A). LGA had a greater mediating effect compared to SGA on the associations of maternal metabolic conditions with NDCs (Table 2 and Fig. 4A). The mediating effect of LGA on the association between PGDM and autism was greater (12.90%) compared to ID (1.73%) and ADHD (2.49%), while none of the NIEs for these relationships were statistically significant after applying the Bonferroni correction for *P* values (*P* < 0.004). The mediating effects of labor complications were generally minimal and not statistically significant for any of the pathways examined. The mediating effects of preterm birth and intrauterine hypoxia were greater for the relationship between PGDM and children’s risks of NDCs compared to the association between GDM or maternal adiposity and children’s NDC outcomes. For PGDM and NDC outcomes, the proportions of the relationship mediated by preterm birth varied from 11.68% for ADHD to 17.78% for ID, while for maternal adiposity and NDC outcomes, the proportions of the relationships mediated by preterm birth varied from 0.67% for ADHD to 2.12% for ID. About PGDM, the proportions of the relationship mediated by intrauterine hypoxia varied from 2.85% for autism to 8.07% for ID, while for maternal adiposity and NDC outcomes, the proportions of the relationships mediated by intrauterine hypoxia varied from 0.57% for ADHD to 1.79% for ID. After applying the Bonferroni correction for *P* values (*P* < 0.004), none of the NIEs related to GDM were statistically significant.

Similarly to prenatal complications, PGDM showed a stronger association with neonatal complications compared to GDM and adiposity (Fig. 2A; Additional file 1: Table S2A). The mediating effects of neonatal complications, such as asphyxia complications, hematological comorbidities, hypoglycemia, and infection were greater for the associations between PGDM and NDCs compared to the association between GDM or adiposity and NDCs (Table 2; Fig. 4A). Notably, the mediating effect of neonatal asphyxia comorbidities on the association between PGDM and ID was greater (18.56%) compared to autism (7.30%) and ADHD (8.34%). The mediating effect of neonatal asphyxia comorbidities on the association with ID was 6.88% for GDM and 5.08% for adiposity. Similarly, the mediating effects of neonatal hematological comorbidities were stronger in associations with PGDM than with GDM and adiposity. For PGDM, these effects were stronger for ID (10.90%) than for autism (7.90%) and ADHD (4.77%).

The mediating effects of GDM for the associations between maternal adiposity and children’s NDC outcomes were generally small, varying from 0.86% for ADHD to 1.16% for autism.

### Joint mediating effects of multiple mediators

The Phi coefficients for mediator correlations ranged from the lowest value of 0.002 (between preterm birth and LGA) to the highest value of 0.397 (between preterm birth and neonatal hematological comorbidities) (*P* < 0.001) (Additional file 1: Figure S2). Following the pattern observed for the individual mediators, the joint mediating effects of all obstetric and neonatal complications for the association between PGDM and children’s NDC outcomes were higher than for the associations between GDM or adiposity and NDCs, though the proportions mediated varied across different NDC diagnoses (Table 2; Fig. 4A). The proportions of the relationship between PGDM and NDCs jointly mediated by obstetric and neonatal complications were highest for ID (44.4%) and autism (42.9%), followed by ADHD (31.4%), compared to the lower proportions of the relationships between high maternal adiposity and ADHD (1.5%), autism (5.3%) or ID (8.1%). While none of the NIEs associated with GDM indicated that joint obstetric and neonatal complications significantly mediated the relationship with offspring NDC, GDM itself had a direct impact on offspring NDCs: NDE (OR 1.25, 95% CI [1.24–1.26] for autism; 1.26 [1.25–1.28] for ID; and 1.14 [1.13–1.14] for ADHD) (Table 2B).

After replicating the multiple mediation analyses with a 99.6% CI, all the joint mediating effects of all complications on associations with PGDM remained statistically significant. For maternal adiposity, the only statistically significant mediating effects were the joint effects of all mediators on ID (8.1%) (Additional file 1: Table S4).

### Joint mediating effects of multiple mediators potentially occurring at different stages in early life

In Table 3 and Fig. 4B, we observed greater joint mediating effects of complications during the neonatal period for the associations of maternal metabolic conditions with different NDCs in offspring, compared to complications during pregnancy and at delivery, particularly for PGDM. For the relationship between PGDM and offspring NDCs, the proportions mediated by complications during the neonatal period varied between 28.1% (ADHD) and 50.1% (ID).

**Table 3:** Multiple mediation analysis of mediators at different stages

	**Stage 1**^**a**^	**Stage 2**^**b**^	**Stage 3**^**c**^
	**OR (95% CI)**	**OR (95% CI)**	**OR (95% CI)**
**A PGDM**			
**Any NDCs**			
NDE	1.37 (1.36–1.38)	1.35 (1.34–1.36)	1.28 (1.27–1.29)
NIE	1.05 (1.01–1.11)	1.07 (1.03–1.13)	1.13 (1.08–1.19)
TE	1.44 (1.38–1.51)	1.44 (1.38–1.51)	1.44 (1.38–1.51)
Proportion mediated, %	14.1**	18.8**	33.1**
**Autism**			
NDE	1.36 (1.35–1.38)	1.33 (1.31–1.34)	1.27 (1.26–1.29)
NIE	1.06 (0.97–1.14)	1.09 (0.99–1.17)	1.13 (1.03–1.22)
TE	1.44 (1.31–1.55)	1.44 (1.31–1.55)	1.44 (1.31–1.55)
Proportion mediated, %	15.1	22.4	34.0**
**ID**			
NDE	1.70 (1.68–1.72)	1.60 (1.57–1.62)	1.33 (1.32–1.35)
NIE	1.05 (0.96–1.15)	1.12 (1.02–1.22)	1.34 (1.22–1.46)
TE	1.77 (1.63–1.96)	1.77 (1.63–1.96)	1.77 (1.63–1.96)
Proportion mediated, %	8.6	19.4*	50.1**
**ADHD**			
NDE	1.34 (1.33–1.35)	1.33 (1.32–1.34)	1.29 (1.28–1.29)
NIE	1.06 (0.99–1.12)	1.06 (0.99–1.13)	1.10 (1.03–1.17)
TE	1.42 (1.32–1.50)	1.42 (1.32–1.50)	1.42 (1.32–1.50)
Proportion mediated, %	15.6	17.7	28.1**
**B GDM**			
**Any NDCs**			
NDE	1.21 (1.20–1.22)	1.22 (1.21–1.22)	1.18 (1.17–1.18)
NIE	0.98 (0.91–1.05)	0.97 (0.91–1.04)	1.01 (0.94–1.08)
TE	1.19 (1.10–1.27)	1.19 (1.10–1.27)	1.19 (1.10–1.27)
Proportion mediated, %	-11.8	-15.6	3.6
**Autism**			
NDE	1.29 (1.28–1.30)	1.29 (1.28–1.30)	1.26 (1.24–1.27)
NIE	1.05 (0.95–1.15)	1.05 (0.95–1.15)	1.08 (0.97–1.18)
TE	1.35 (1.22–1.48)	1.35 (1.22–1.48)	1.35 (1.22–1.48)
Proportion mediated, %	15.8	15.8	24.1
**ID**			
NDE	1.32 (1.30–1.34)	1.34 (1.32–1.36)	1.21 (1.19–1.23)
NIE	1.02 (0.92–1.21)	1.01 (0.91–1.19)	1.12 (1.01–1.32)
TE	1.35 (1.21–1.60)	1.37 (1.21–1.60)	1.35 (1.21–1.60)
Proportion mediated, %	7.6	2.7	37.6*
**ADHD**			
NDE	1.15 (1.14–1.16)	1.16 (1.15–1.17)	1.13 (1.12–1.39)
NIE	0.97 (0.89–1.09)	0.96 (0.88–1.08)	0.99 (0.91–1.11)
TE	1.12 (1.03–1.25)	1.12 (1.03–1.25)	1.12 (1.03–1.25)
Proportion mediated, %	-26.5	-34.5	-11.6
**C Adiposity**			
**Any NDCs**			
NDE	1.36 (1.34–1.37)	1.36 (1.35–1.37)	1.35 (1.33–1.36)
NIE	1.00 (0.99–1.01)	1.00 (0.99–1.01)	1.01 (1.00–1.02)
TE	1.36 (1.34–1.38)	1.36 (1.34–1.38)	1.36 (1.34–1.38)
Proportion mediated, %	0.8	0.1	2.9
**Autism**			
NDE	1.38 (1.36–1.40)	1.38 (1.36–1.40)	1.37 (1.35–1.39)
NIE	0.99 (0.97–1.01)	0.99 (0.97–1.01)	0.99 (0.97–1.01)
TE	1.36 (1.33–1.39)	1.36 (1.33–1.39)	1.36 (1.33–1.39)
Proportion mediated, %	-4.1	-4.5	-2.5
**ID**			
NDE	1.58 (1.55–1.62)	1.59 (1.56–1.62)	1.53 (1.50–1.57)
NIE	0.99 (0.97–1.02)	0.99 (0.97–1.01)	1.03 (1.00–1.05)
TE	1.57 (1.51–1.63)	1.57 (1.51–1.63)	1.57 (1.51–1.63)
Proportion mediated, %	-1.4	-2.0	5.5*
**ADHD**			
NDE	1.37 (1.36–1.38)	1.38 (1.36–1.39)	1.37 (1.35–1.38)
NIE	1.00 (0.98–1.01)	0.99 (0.98–1.01)	1.00 (0.99–1.01)
TE	1.37 (1.34–1.39)	1.37 (1.34–1.39)	1.37 (1.34–1.39)
Proportion mediated, %	-1.3	-2.0	-0.1

All analyses were adjusted for the child’s sex, birthyear, maternal age, birth order, maternal birth country, disposable income at birth, and maternal psychiatric history. Analyses for GDM were additionally adjusted for maternal BMI. Mediated proportions were marked with “*” when the 95% CI for NIEs did not encompass a value of 1, and by “**” when the 99.6% CI for NIEs did not encompass a value of 1 (refer to Additional file 1: Table S4), with both NDE and NIE having the same direction

The rationale for including different mediators in each multiple mediation analysis was described in Additional file 1: Figure S1

^a^Mediators in stage 1 for PGDM and GDM included: pregnancy hypertensive diseases, placenta/membrane disorders, and gestational infection

Mediators in stage 1 for adiposity include: pregnancy hypertensive diseases, placenta/membrane disorders, gestational infection, and GDM

^b^Mediators in stage 2 included: SGA, and LGA, intrauterine hypoxia, labour complications, and preterm birth

^c^Mediators in stage 3 included: neonatal asphyxia comorbidities, neonatal hematological comorbidities, neonatal hypoglycemia, and neonatal infection

After replicating the multiple mediation analyses with a 99.6% CI, most joint mediating effects of complications at different stages on associations with PGDM remained statistically significant, except for the joint mediating effects of complications at stage 2 on ID. No mediating effects of complications at different stages on associations with GDM or maternal adiposity remained statistically significant (Additional file 1: Table S4).

### Sensitivity analysis

When maternal T1DM diagnoses were specifically considered instead of any PGDM diagnosis, we observed similar results compared to the analysis considering potential mediators for the relationship between any PGDM children’s NDC outcomes (Additional file 1: Table S5). The results remained similar to the results in the main analysis when restricting the study sample to those born in 1997 and onwards (Additional file 1: Table S6) or randomly selecting one child from each mother (Additional file 1: Table S7).

## Discussion

### Main findings

In this Swedish register-based cohort study, we found evidence for the joint mediating effects of obstetric and neonatal complications on the relationships between maternal metabolic conditions and offspring NDCs. The proportions mediated by obstetric and neonatal complications were higher for the relationships between PGDM and children’s NDC outcomes compared to the relationships between GDM or maternal overweight/obesity and children’s NDC outcomes. Most complications exhibited minor individual mediating effects. However, the mediating effects of complications such as pregnancy hypertensive diseases, preterm birth, neonatal asphyxia, and hematological comorbidities were modest yet stronger compared to other complications examined, such as labor complications or gestational infections. This was especially evident in the associations involving PGDM, where the individual mediating effects were greater than 10%. Additionally, we observed that complications during the neonatal period had relatively greater joint mediating effects compared to complications diagnosed during pregnancy and childbirth periods, especially in the relationships with PGDM.

### Interpretation of main findings

While our study presents novel results about quantifying the mediating effects of obstetric and neonatal complications on the relationship between three common maternal metabolic conditions and offspring autism, ID, and ADHD, our findings, regarding the associations between maternal metabolic conditions and obstetric/neonatal complications, as well as between these complications and offspring NDCs, largely align with prior findings. For example, in a hospital-based cohort study, Billionnet et al. found that different types of maternal diabetes (T1DM, T2DM, and GDM) were associated with a range of obstetric and neonatal complications, including eclampsia/pre-eclampsia, preterm birth, macrosomia, and neonatal asphyxia [11]. A meta-analysis including 39 studies indicated that maternal overweight/obesity was associated with increased risks of several adverse pregnancy outcomes including LGA, preterm birth, and neonatal asphyxia [17], of a similar magnitude and direction to those that we observed here.

In another meta-analysis including 40 studies that examined the associations between perinatal and neonatal complications and autism [27], the authors reported stronger associations between labor complications and the relative risk of autism than we observed in our study. For example, the meta-analysis presented a summarized relative risk estimate of 1.77 (95% CI: 0.76–4.14) for prolonged labor and 4.90 (95% CI: 1.41–16.94) for birth injury or trauma. In contrast, we observed an OR of 1.04 (95% CI: 0.99–1.09) for autism after exposure to labor complications. We detected an association between LGA and odds of autism, while the prior meta-analysis did not find sufficient evidence of an association. However, we observed relationships of similar magnitudes and directions for multiple other complications (placenta/membrane disorders, neonatal asphyxia comorbidities, and preterm birth) and autism. Our findings are also in agreement with previously reported relationships between perinatal complications and ADHD [29], including hypertensive disorders during pregnancy and preterm birth, as well as relationships reported between complications such as low birth weight, preterm birth, and hypertensive conditions and ID [31]. Given that mediation analyses are most suited to situations in which there is a well-defined, well-supported hypothesis regarding the proposed mechanisms to be explored as potential mediating pathways [45], the overall agreement between our observations in the study population and the existing literature is reassuring.

In line with previous studies, the association of maternal PGDM with several obstetric and neonatal complications (e.g., eclampsia/pre-eclampsia, preterm birth, and neonatal asphyxia) were greater compared to maternal GDM and overweight/obesity [11, 50] which partially explained the stronger joint mediating effect of obstetric and neonatal complications in the association between PGDM and NDCs seen in our study. These complications may exert independent or combined effects on the etiology of offspring NDCs, and outcomes following exposure also depend heavily on their timing of occurrence relative to when different neuronal processes or components of human brain development take place [32, 33]. In our study, we found that the joint mediating effects of complications were more relevant to neonatal complications compared to the complications during pregnancy and at birth, especially in the associations between PGDM and NDCs. At this stage, the immature brain is susceptible and vulnerable to environmental influences which subsequently influence the establishment of functionally organized cortical circuits through their potential damage in terms of the development of gray and white matter, neuronal myelination, synaptogenesis, pruning, and synaptic modification [32]. Differences in brain circuits and volume have been frequently reported in ASD [4] and ADHD [3] compared to typically developing children.

While we were able to detect significant mediating effects of obstetric and neonatal complications in the relationship between maternal overweight/obesity and offspring risk of NDCs, each of the mediating proportions was small, at 5% or less for the mediating effects of the individual complications and less than 10% for the joint mediating effects of the combined complications. Previous studies employing genetically informed designs have indicated that the relationship between elevated maternal BMI and children’s risk of NDC outcomes may be explained to a large extent by confounding by shared genetic liabilities between elevated BMI and NDCs [7–9]. For example, sibling comparisons have generally failed to replicate the relationships between maternal BMI and children’s risk of autism and ADHD observed in comparisons of non-related individuals [7–9]. A large proportion mediated by plausible pathways would add to evidence of causal relationships between exposure and outcome, while the relatively small mediation proportions that we observed here for the relationships between elevated maternal BMI and children’s risk of NDC outcomes indicate that the theoretically plausible pathways related to obstetric complications could explain only a very limited proportion of the observed associations. Similar to the sibling comparison studies, this lends support to the notion that the association between elevated maternal BMI and children’s risk of NDCs may be due to a large extent to unobserved confounders.

### Clinical implications

We observed that the mediating effects of pregnancy hypertensive diseases (for autism), preterm birth (for autism, ADHD, and ID), neonatal asphyxia (for ID), and hematological comorbidities (for ID) accounted for more than 10% of the observed relationships between PGDM and specific offspring NDCs. Previous evidence suggested that early intervention strategies can positively influence cognitive and motor outcomes in preterm infants during infancy, with cognitive benefits lasting until preschool age [51]. Clinical trial evidence indicated that preventing neonatal asphyxia through resuscitation positively impacts cognitive and psychomotor outcomes in children up to the age of three [52]. Furthermore, research indicated that preventing neonatal anemia with iron supplementation within the first month after birth can enhance the psychomotor development of vulnerable infants [53]. Aligned with prior studies, our findings provided additional evidence that interventions targeting the aforementioned mediators could modestly mitigate the risk of autism, ADHD, and ID in offspring exposed to maternal metabolic conditions, particularly those exposed to maternal PGDM.

The pronounced joint mediating effects of obstetric and neonatal complications on the relationship between PGDM and children’s NDC outcomes (approximately 40%)—as compared to GDM or maternal adiposity—underscored the importance of comprehensive care for women with PGDM and their children. Conversely, given the minimal mediating effects of obstetric and neonatal complications in women with GDM and overweight/obesity, this emphasized the need to manage these core metabolic conditions effectively. Furthermore, our findings showed that complications during the neonatal period exerted a stronger mediating effect on the associations between maternal metabolic conditions, notably PGDM, and offspring NDC outcomes than did complications during pregnancy or delivery. Given this, neonates with maternal PGDM exposure warranted specialized monitoring and comprehensive care to reduce the risks for NDCs.

### Strengths and limitations

To our knowledge, this is the first study quantifying the mediating effect of obstetric and neonatal complications in the associations between maternal metabolic conditions and NDCs in offspring. Utilizing Swedish registries, we acquired a large study sample size to conduct the mediation analysis. Moreover, access to these registries enabled us to account for a range of potential confounders. Furthermore, biases attributed to potential variance in disease coverage in ICD-9 (1987–1996) and ICD-10 (1997–2018), and an improved ascertainment of NDC cases over time, appear to be minimal. We were also able to rule out potential biases resulting from including non-independent observations of multiple siblings born to the same mother.

Traditional approaches to mediation analysis are based on structural equations [54–56]. For example, the Baron and Kenny approach compares the coefficients in two standard regressions, one adjusted for the mediator and the other non-adjusted [55]. However, these traditional approaches cannot immediately extend to discrete mediators and outcomes [46]. In addition, they cannot accommodate situations where the exposure-outcome relationship is not linear. Most importantly, traditional approaches do not allow for the interaction between exposure and mediator [47, 57, 58]. The methods used in this study address the above-mentioned limitations: we were able to evaluate the mediation effect of binary mediators for binary outcome measures without assuming linear relationships; we also allowed for potential interactions between maternal metabolic conditions and obstetric outcomes, which have been previously suggested [59, 60]. Furthermore, we incorporated multiple mediation analyses, which allows for the plausible situation in which the presence of one obstetric or delivery complication can influence the likelihood of another and also enabled us to estimate the joint mediating effect of multiple complications which often co-occur.

However, our study must be interpreted in light of several limitations. First, though we have considered several potential confounders, results of direct and indirect effects could still be biased due to unmeasured confounders [47, 49, 61], such as genetic factors. However, we expect those factors to affect the mediators and outcomes in the same direction (i.e., a genetic factor would increase the risk for adverse obstetric outcome and also increase the risk for offspring autism).VanderWeele and colleagues have suggested that this type of unmeasured confounder is likely to cause underestimated direct effect and overestimated indirect effect [61, 62]. Secondly, as most cases of PGDM in our study were T1DM, the results for PGDM are largely driven by T1DM. We were unable to perform mediation analyses for the associations between T2DM and NDCs due to limited cases of exposure and outcomes when taking mediators into account. Thirdly, in Sweden, the prevalence of GDM appeared lower than in other high-income countries [63]. Throughout the study period, inconsistencies in GDM diagnostic criteria among Swedish regions were observed [63]. Swedish clinicians and researchers suggested that the actual GDM prevalence might be underestimated within the country [63, 64]. As the number of cases of GDM was relatively small, our power to quantify mediating relationships between GDM and children’s risk of NDC outcome might also be limited. Additionally, this non-differential measurement error of exposure can cause the natural direct effect to be biased toward the null, while the indirect effect may be biased in either direction [45]. Fourthly, we utilized maternal BMI at the first antenatal care visit as a proxy for maternal pregestational BMI, which did not consider the variation in the week of measurement. However, in Sweden, approximately 90% of women had their initial antenatal care visit around 9–10 weeks of gestational age [7, 41, 42], and weight gain during the first trimester (< 14 weeks of gestational age) is minimal, ranging from 0.5 to 2 kg [65]. Hence, the influence of variation in the timing of the first BMI measurement within the first trimester was considered to be minimal, though this could still introduce a small degree of misclassification into the baseline BMI categories. Fifthly, we categorized our mediators based on periods—during pregnancy, at birth, and in the neonatal period—rather than relying on specific diagnosis dates. This approach was chosen due to potential discrepancies or delays between the true onset date, the diagnostic date, and the recording date. As a result, when mediators appear within the same timeframe, bidirectional relationships, such as those between GDM and pregnancy hypertensive diseases, might develop. In the case where such bidirectional relationships may be present, our results should be interpreted with caution as the proportion mediated may be inflated. However, we observed a generally small proportion of the relationship between GDM and offspring NDCs mediated. Sixthly, this study spanned three decades, during which the diagnostic criteria for exposures, mediators, and outcomes might have changed. Thus, we adjusted for birthyear in the models. Additionally, we conducted a sensitivity analysis on individuals born after 1997, the year the ICD-10 was introduced, and our primary findings remained consistent. Nevertheless, to bolster the validity of our conclusions, future studies using more up-to-date datasets are warranted. Lastly, while we have examined a broad spectrum of obstetric and neonatal complications in this study, we did not assess the mediating effects of preventive measures (e.g., cesarean section). Such evaluations are recommended for future research.

## Conclusions

In summary, the observed associations between maternal metabolic conditions and increased risks of NDCs in offspring may be influenced by subsequent obstetric and neonatal complications. Addressing complications such as pregnancy hypertensive diseases, preterm birth, neonatal asphyxia, and hematological comorbidities and enhancing the management of neonatal complications could potentially mitigate the risk of offspring NDCs, especially in the context of maternal PGDM exposure. The modest mediating effects of obstetric and neonatal complications in the relationships between GDM, overweight/obesity, and offspring NDCs underscore the importance of addressing the primary metabolic factors in addition to managing complications.

### Supplementary Information

**Additional file 1:** **Figure S1. **Potential pathways linking maternal metabolic conditions and NDCs in offspring. **Figure S2. **Correlations between mediators. **Figure S3. **Illustration of multiple mediation analysis using a Weight-Based Approach. **Table S1. **Codes and definitions for exposures, outcomes, and mediators. **Table S2. **Associations between maternal metabolic conditions, obstetric and neonatal complications, and offspring NDCs. **Table S3.** Characteristics of the study sample over outcomes. **Table S4.** Multiple mediation analysis of the association between maternal metabolic conditions and NDCs in offspring: odds ratios with 99.6% confidence intervals. **Table S5.** Sensitivity analysis for the single mediation analysis in the association between T1DM and NDCs in offspring. **Table S6.** Sensitivity analysis for the single mediation analysis of the association between maternal adverse metabolic conditions and any NDCs in offspring born no earlier than 1997. **Table S7.** Sensitivity analysis for the single mediation analysis, derived by randomly selecting one child from each mother.

## Data Availability

Data used for the current study were pseudonymized and obtained from Statistics Sweden and the National Board of Health and Welfare after ethical and legal assessment. Researchers interested in obtaining the data and replicating our results can make inquiry through these data holders. For further information, see: https://www.scb.se/en/services/guidance-for-researchers-and-universities/.

## Abbreviations

PGDM: Pregestational diabetes
GDM: Gestational diabetes mellitus
NDCs: Neurodevelopmental conditions
ADHD: Attention-deficit/ hyperactivity disorder
ID: Intellectual disabilities
SGA: Small for gestational age
LGA: Large for gestational age
OR: Odds ratio
CI: Confidence interval
NDE: Natural direct effect
NIE: Natural indirect effect
TE: Total effect
