# Supplementary material for: Associations between maternal metabolic conditions and neurodevelopmental conditions in offspring: the mediating effects of obstetric and neonatal complications
Source: BMC Med. 2023 Nov 7;21:422. doi: 10.1186/s12916-023-03116-x (PMC10631144; doi:10.1186/s12916-023-03116-x)
Supplement: Supplementary file 1 — Additional file 1: Figure S1. Potential pathways linking maternal metabolic conditions and NDCs in offspring. Figure S2. Correlations between mediators. Figure S3. Illustration of multiple mediation analysis using a Weight-Based Approach. Table S1. Codes and definitions for exposures, outcomes, and mediators. Table S2. Associations between maternal metabolic conditions, obstetric and neonatal complications, and offspring NDCs. Table S3. Characteristics of the study sample over outcomes. Table S4. Multiple mediation analysis of the association between maternal metabolic conditions and NDCs in offspring: odds ratios with 99.6% confidence intervals. Table S5. Sensitivity analysis for the single mediation analysis in the association between T1DM and NDCs in offspring. Table S6. Sensitivity analysis for the single mediation analysis of the association between maternal adverse metabolic conditions and any NDCs in offspring born no earlier than 1997. Table S7. Sensitivity analysis for the single mediation analysis, derived by randomly selecting one child from each mother. [file 12916_2023_3116_MOESM1_ESM.docx]

**Additional file 1**

**Figure S1.** Potential pathways linking maternal metabolic conditions and NDCs in offspring. Mediators were chronologically categorized into three stages: the placental stage, the fetal growth and birth stage, and the neonatal stage. Each mediator can manifest independently or coexist with other downstream mediators, depending on the severity of maternal adverse metabolic conditions and clinical management.

**
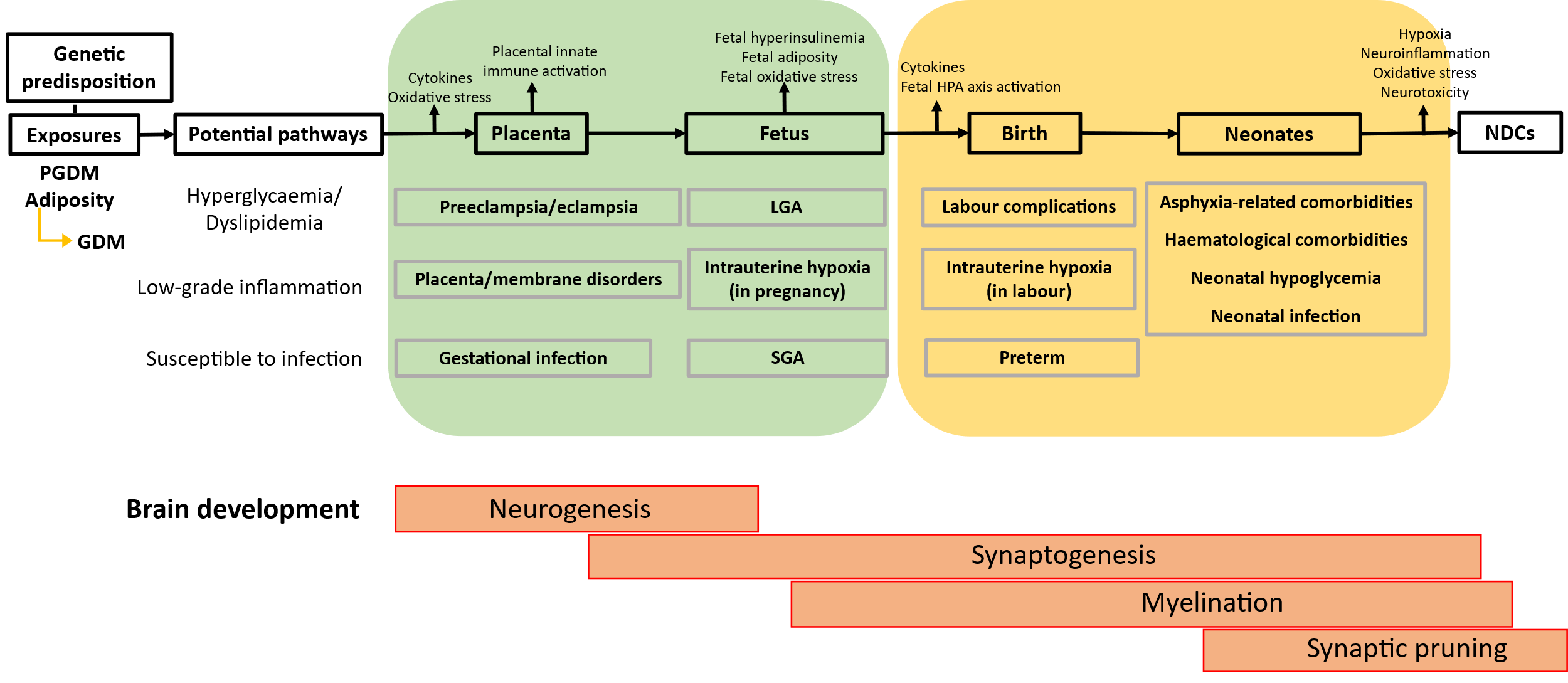
**

**Figure S2.** Correlations between mediators

Correlations were calculated using the Phi coefficient, a measure derived from the chi-squared statistic, suitable for assessing associations between binary variables.

**Figure S3.** Illustration of multiple mediation analysis using a Weight-Based Approach.
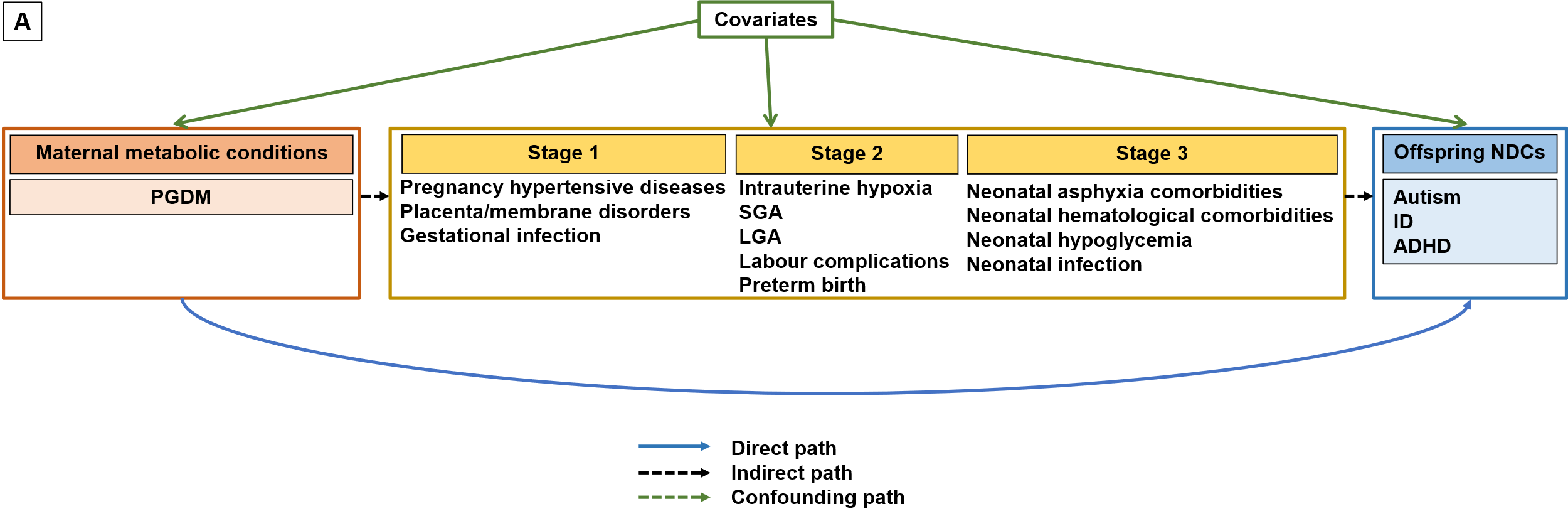


**
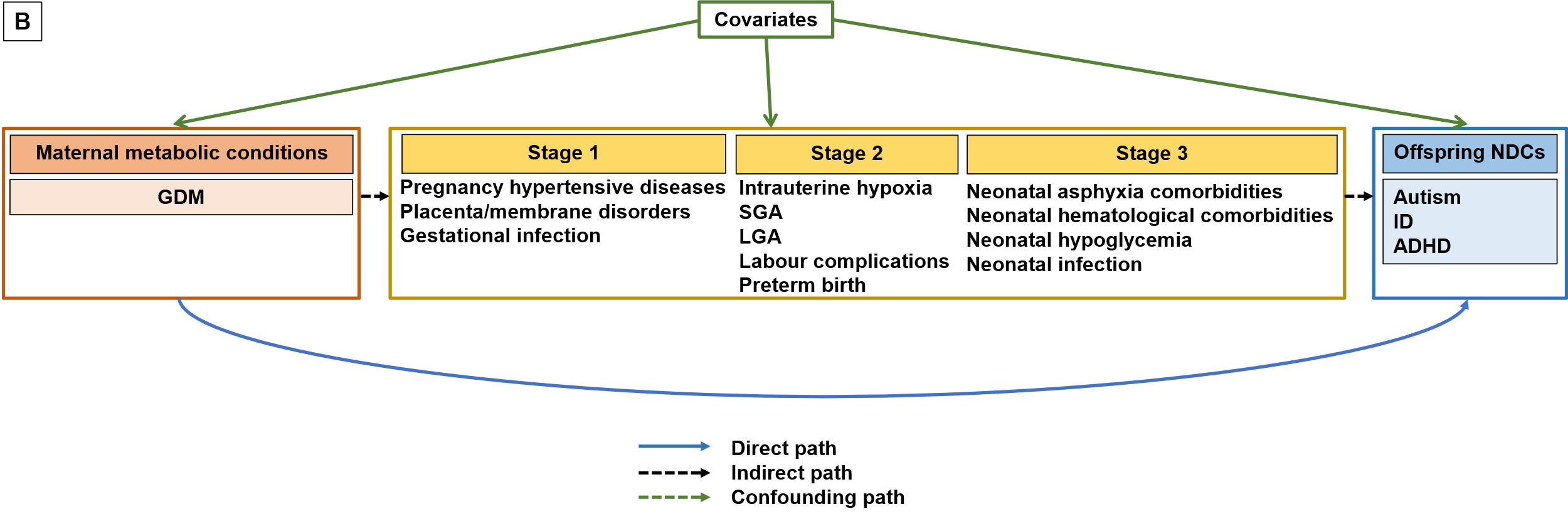
**

**
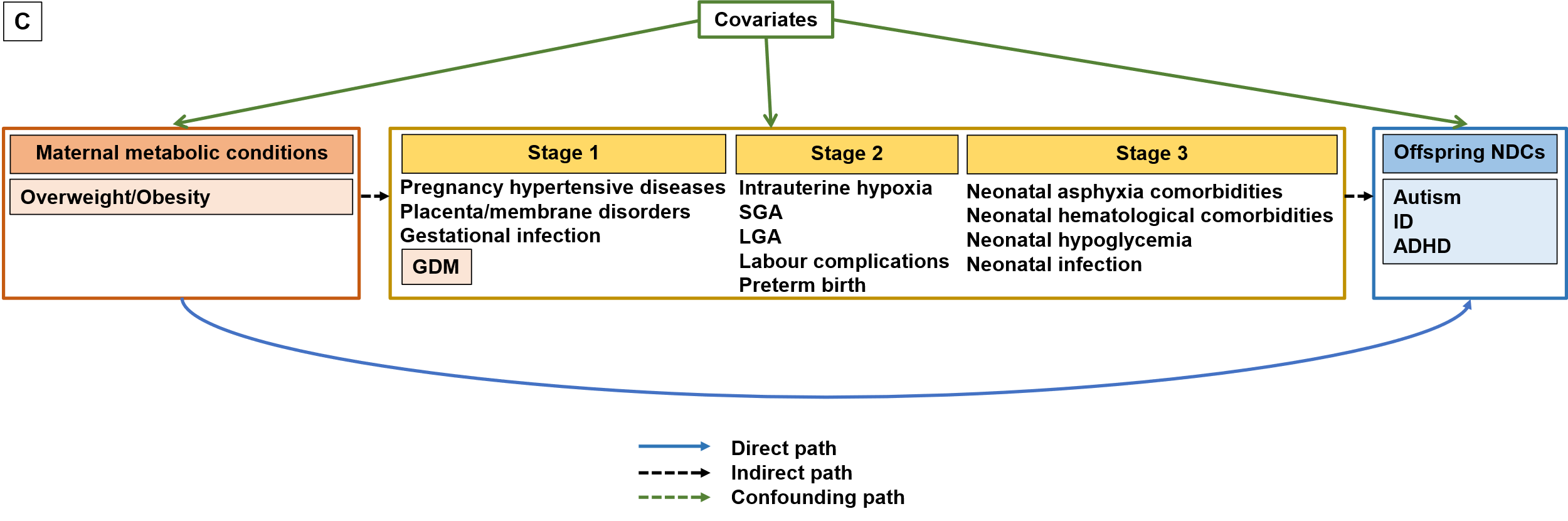
**

**Notations:**

i: individual in the study sample

**Panel A:**

A: exposure variable-PGDM

a: exposed---PGDM

a*: unexposed---without PDGM or GDM

P(A=a): the proportion of PGDM in the population

P(A=a*): the proportion of normal group (without PGDM/GDM) in the population

Ai: individual’s actual exposure value

**Panel B:**

A: exposure variable-GDM

a: exposed---GDM

a*: unexposed---without GDM or PGDM

P(A=a): the proportion of GDM in the population

P(A=a*): the proportion of normal group (without GDM/PGDM) in the population

Ai: individual’s actual exposure value

**Panel C:**

A: exposure variable-overweight/obesity

a: exposed---overweight/obesity

a*: unexposed---normal BMI

P(A=a): the proportion of overweight/obesity in the population

P(A=a*): the proportion of normal group (with normal BMI) in the population

Ai: individual’s actual exposure value

M: a set of multiple mediators (shown in the ***panel A, B and C***)

Mi: individual’s actual mediators’ values

M_a_: potential values of mediators if A was set to a

M_a*_: potential values of mediators if A was set to a*

Y: outcome variable---offspring NDC diagnosis

E[Y]: potential values of the outcome

C: a set of covariates

C_i_: everyone’s actual covariates values

**Step I:** Fit a logistic regression model for the mediator: logit (A=a) = C

Using this model, calculate every individual’s probability of being exposed giving his covariates P(A=a|ci). Also, calculate P(A=a*|ci) = 1 - P(A=a|ci).

**Step II:** Fit a logistic regression model for the outcome: logit (Y)=A+M+C

For each individual i with Ai=a*, use this model to obtain a predicted estimate of Y if the individual had Ai=a rather than Ai=a*. This is **E(Y|A=a, M=Mi, C=Ci).**

**Step III:** Calculate three potential values of the outcome

• E[Y_aMa_]= weighted average of Y among individuals with Ai=a, where the weight is P(A=a)/P(A=a|ci)

• E[Y_a*Ma*_] = weighted average of Y among individuals with Ai=a*, where the weight is P(A=a*)/P(A=a*|ci)

• E[Y_aMa*_] = weighted average of E(Y|A=a, M=Mi, C=Ci) among individuals with Ai=a*, where the weight is P(A=a*)/P(A=a*|ci)

**Step IV:** Calculate mediation effect estimates:

- Natural direct effect (NDE)=: E[Y_aMa*_]- E[Y_a*Ma*_]
- Natural indirect effect (NIE)= E[Y_aMa_]- E[Y_aMa*_]
- Total effect (TE)= E[Y_aMa_]- E[Y_a*Ma*_]

**Table S1.** Codes and definitions for exposures, outcomes, and mediators.

| **Variables** | **Disease name** | **ICD-9^ab^** | **ICD-10^ab^** | **ICD-8^ab^** | **Prescription Drug Register^c^** |
| --- | --- | --- | --- | --- | --- |
| Pregnancy hypertensive diseases | Pre-eclampsia | 642E, 642F, 642X | O13, O14 |  |  |
|  | Eclampsia | 642G, 642H | O15 |  |  |
| Placenta/membrane disorders | Placental disorders (Malformation of placenta (Abnormal placenta NOS, Circumvallate placenta); Other placental disorders (Placental: dysfunction, infarction) | 656F, 656H, 762C | O43.1, O43.8, O36.5, P02.2 |  |  |
|  | Premature rupture of membranes | 658B | O42 |  |  |
|  | Premature separation of placenta [abruptio placentae] | 641C, 762B | O45, P02.1 |  |  |
|  | Antepartum haemorrhage, not elsewhere classified | 641C, 641D, 641W, 641X | O46 |  |  |
| Gestational infection | Infection of amniotic sac and membranes/ Chorioamnionitis | 658E, 762H | O41.1, P02.7 |  |  |
|  | Infections of genitourinary tract | 646F, 646G | O23 |  |  |
|  | Other gestational infections | Refer to PMID: 26303935. | |  |  |
| Labour complications | Long labour | 662A, 662B, 662C | O63 (O63.0, O63.1, O63.9) |  |  |
|  | Other obstructed labour | 660E, 660G, 660H, 660W, 660X | O66.0, O66.2, O66.4, O66.5, O66.8, O66.9 |  |  |
|  | Birth trauma (newborn) | 767A, 767B, 767C, 767D, 767E, 767F, 767G,767H, 767W, 767X, 772B, 772C | P10-P15 |  |  |
| Intrauterine hypoxia | Intrauterine hypoxia | 768C, 768D, 768E, 656D | P20, O36.3 |  |  |
|  | Labour and delivery complicated by fetal stress [distress] | 656D | O68 |  |  |
| Neonatal asphyxia comorbidities | Birth asphyxia | 768F, 768G, 768X, 770W | P21 |  |  |
|  | Neonatal aspiration syndromes | 770B | P24 |  |  |
|  | Respiratory distress of newborn | 769X,770G,770W | P22 |  |  |
|  | Convulsions | 779A | P90 |  |  |
|  | Hypoxic ischemic encephalopathy and related conditions | 779B, 779C | P910, P913-P916, P918, P919 |  |  |
| Neonatal hematological comorbidities | Neonatal jaundice | 773A-F, 774B-H | P55-P59 |  |  |
|  | Neonatal anemia | 776F, 776G, 280X, 285X | P61.2, P61.3, P61.4, D50, D64.9 |  |  |
|  | Polycythaemia neonatorum | 776E | P61.1 |  |  |
| Neonatal infection |  | Refer to PMID: 24366719 | |  |  |
| Neonatal hypoglycemia | Neonatal hypoglycemia | 775G | P70.3, P70.4 |  |  |
| PGDM | Type 1 diabetes |  | E10, O24.0 |  |  |
|  | Type 2 diabetes |  | E11, 024.1 |  |  |
|  | PGDM-not specified^d^ | 250, 648A, 790C |  | 250 |  |
| GDM | GDM | 648W | O24.4 |  |  |
| ASD |  | 299 | F84 |  |  |
| ADHD |  | 314 | F90 |  | methylphenidate [N06BA04] or atomoxetin [N06BA09] |
| ID |  | 317-319 | F70-F79 |  |  |
| Maternal psychiatric history |  | 290-319 | F chapter | 290-315 |  |
|  |  |  |  |  |  |
| **Other mediators** | **Definition** | | | | |
| SGA | **Male**  **Mean (birthweight)** = (-(1.907345*10^(-6))*days of gestation^4 + (1.140644*10^(-3))*days of gestation^3 -0.1336265*days^2 +1.976961*days of gestation+241.0053)  **z-score**= (birthweight – mean [birthweight])/(0.12 * mean [birthweight])  **Female**  **Mean (birthweight)=** (-(2.761948*10^(-6))* days of gestation ^4 + (1.744841*10^(-3))* days of gestation s^3 -0.2893623* days of gestation^2 + 18.91197* days of gestation-413.5122)  **z-score**= (birthweight – mean [birthweight])/(0.12 * mean [birthweight])  **SGA: z-score<-2** | | | | |
| LGA | **LGA: z-score>2** | | | | |
| Preterm birth | Gestational week at birth<37 weeks | | | | |

^a^The National Patient Register (NPR): including inpatient care beginning in 1973, outpatient physician visits in specialist care beginning in 1997, and outpatient psychiatric diagnoses from 2006, children and adolescent psychiatric care (2011)

^b^The Medical Birth Register (MBR): including information from medical records from prenatal, delivery, and neonatal healthcare beginning in 1973.

^c^The Prescribed Drug Register (PDR) contains data on Anatomical Therapeutic Chemical (ATC) classification codes for medications dispensed to the entire population in Sweden since 1 July 2005. Receipt of a prescription for attention-deficit/hyperactivity disorder (ADHD) medications is a useful proxy for an ADHD diagnosis, as Swedish medical guidelines mandate that ADHD medications should only be prescribed by a psychiatric specialist and after other (non-pharmacological) interventions have failed.

^d^If only ICD-8 or ICD-9 codes were recorded before the birth of the index person, we searched for maternal ICD-10 diagnoses of Type I and Type II diabetes mellitus until the end of follow-up (December 31, 2016). If the mother had an ICD-10 coded Type I or Type II diagnosis recorded after the birth of the index person (in addition to an ICD-8 or ICD-9 code recorded before the birth of the index person), we assigned the mother accordingly as having Type I or Type II pregestational diabetes mellitus. If the mother did not have an ICD-10 coded Type I or Type II diabetes mellitus diagnosis after the birth of the index person but received the first ICD-8 or ICD-9 250 diagnosis before her 18th birthday, we assigned the mother to the Type I diabetes mellitus category. If the mother received an ICD-8 or ICD-9 250 diagnosis before the birth of the index person but could not be confidently assigned as having a Type I or Type II diagnosis, we assigned the mother to the category of “Pregestational Diabetes mellitus - Not Otherwise Specified”.

**Table S2.** Associations between maternal metabolic conditions, obstetric and neonatal complications, and offspring NDCs

**A Associations of maternal metabolic conditions with obstetric and neonatal complications^a^**

|  | **PGDM** |  | **GDM** |  | **Adiposity** |  |
| --- | --- | --- | --- | --- | --- | --- |
|  | **OR (95% CI)** | **P-value** | **OR (95% CI)** | **P-value** | **OR (95% CI)** | **P-value** |
| **Pregnancy hypertensive diseases** | 3.70 (3.55 - 3.85) | <0.001 | 1.87 (1.78 - 1.96) | <0.001 | 2.28 (2.25 - 2.32) | <0.001 |
| **Placenta/membrane disorders** | 1.51 (1.44 - 1.59) | <0.001 | 1.25 (1.19 - 1.33) | <0.001 | 0.95 (0.93 - 0.96) | <0.001 |
| **Maternal Infection** | 2.22 (2.11 - 2.34) | <0.001 | 1.41 (1.33 - 1.50) | <0.001 | 1.16 (1.14 - 1.18) | <0.001 |
| **Intrauterine hypoxia** | 2.04 (1.96 - 2.13) | <0.001 | 1.05 (1.00 - 1.10) | 0.076 | 1.24 (1.22 - 1.25) | <0.001 |
| **SGA** | 1.13 (1.03 - 1.24) | 0.010 | 0.78 (0.71 - 0.87) | <0.001 | 0.93 (0.91 - 0.95) | <0.001 |
| **LGA** | 7.72 (7.46 - 8.00) | <0.001 | 3.34 (3.20 - 3.49) | <0.001 | 2.42 (2.38 - 2.46) | <0.001 |
| **Labour complications** | 1.86 (1.76 - 1.97) | <0.001 | 1.25 (1.17 - 1.34) | <0.001 | 1.27 (1.25 - 1.30) | <0.001 |
| **Preterm birth** | 3.37 (3.24 - 3.50) | <0.001 | 1.65 (1.57 - 1.74) | <0.001 | 1.19 (1.17 - 1.20) | <0.001 |
| **Neonatal asphyxia comorbidities** | 3.03 (2.91 - 3.17) | <0.001 | 1.42 (1.34 - 1.50) | <0.001 | 1.39 (1.37 - 1.41) | <0.001 |
| **Neonatal hematological comorbidities** | 2.82 (2.71 - 2.94) | <0.001 | 1.52 (1.45 - 1.60) | <0.001 | 1.31 (1.29 - 1.33) | <0.001 |
| **Neonatal hypoglycemia** | 12.20 (11.73 - 12.69) | <0.001 | 4.60 (4.37 - 4.85) | <0.001 | 1.60 (1.56 - 1.63) | <0.001 |
| **Neonatal infection** | 1.75 (1.65 - 1.86) | <0.001 | 1.28 (1.20 - 1.37) | <0.001 | 1.20 (1.18 - 1.22) | <0.001 |

**B Associations of obstetric and neonatal complications with offspring NDCs^b^**

|  | **Any NDCs** |  | **Autism** |  | **ID** |  | **ADHD** |  |
| --- | --- | --- | --- | --- | --- | --- | --- | --- |
|  | **OR (95% CI)** | **P-value** | **OR (95% CI)** | **P-value** | **OR (95% CI)** | **P-value** | **OR (95% CI)** | **P-value** |
| **Pregnancy hypertensive diseases** | 1.25 (1.22- 1.28) | <0.001 | 1.30 (1.25 - 1.35) | <0.001 | 1.60 (1.51 - 1.70) | <0.001 | 1.20 (1.17 - 1.24) | <0.001 |
| **Placenta/membrane disorders** | 1.40 (1.37 - 1.43) | <0.001 | 1.32 (1.27 - 1.37) | <0.001 | 2.29 (2.18 - 2.40) | <0.001 | 1.30 (1.27 - 1.34) | <0.001 |
| **Maternal Infection** | 1.34 (1.30 - 1.37) | <0.001 | 1.31 (1.25 - 1.37) | <0.001 | 1.48 (1.39 - 1.58) | <0.001 | 1.34 (1.30 - 1.38) | <0.001 |
| **Intrauterine hypoxia** | 1.21 (1.19 - 1.24) | <0.001 | 1.15 (1.12 - 1.19) | <0.001 | 1.70 (1.62 - 1.78) | <0.001 | 1.15 (1.12 - 1.18) | <0.001 |
| **SGA** | 1.73 (1.68 - 1.79) | <0.001 | 1.64 (1.56 - 1.73) | <0.001 | 3.48 (3.30 - 3.67) | <0.001 | 1.54 (1.48 - 1.59) | <0.001 |
| **LGA** | 1.10 (1.07 - 1.13) | <0.001 | 1.22 (1.16 - 1.28) | <0.001 | 1.14 (1.06 - 1.23) | <0.001 | 1.07 (1.04 - 1.11) | <0.001 |
| **Labour complications** | 1.06 (1.03 - 1.10) | <0.001 | 1.04 (0.99 - 1.09) | 0.148 | 1.26 (1.18 - 1.35) | <0.001 | 1.02 (0.99 - 1.06) | 0.191 |
| **Preterm birth** | 1.44 (1.41 - 1.47) | <0.001 | 1.40 (1.35 - 1.45) | <0.001 | 2.46 (2.35 - 2.57) | <0.001 | 1.32 (1.29 - 1.36) | <0.001 |
| **Neonatal asphyxia comorbidities** | 1.52 (1.48 - 1.55) | <0.001 | 1.46 (1.40 - 1.51) | <0.001 | 3.30 (3.17 - 3.44) | <0.001 | 1.27 (1.23 - 1.31) | <0.001 |
| **Neonatal hematological comorbidities** | 1.35 (1.32 - 1.38) | <0.001 | 1.32 (1.27 - 1.37) | <0.001 | 2.06 (1.96 - 2.16) | <0.001 | 1.26 (1.22 - 1.29) | <0.001 |
| **Neonatal hypoglycemia** | 1.64 (1.59 - 1.70) | <0.001 | 1.65 (1.56 - 1.74) | <0.001 | 3.20 (3.00 - 3.41) | <0.001 | 1.46 (1.41 - 1.52) | <0.001 |
| **Neonatal infection** | 1.30 (1.26 - 1.34) | <0.001 | 1.34 (1.28 - 1.40) | <0.001 | 2.01 (1.89 - 2.12) | <0.001 | 1.20 (1.16 - 1.24) | <0.001 |

Logistic regression, with standard errors adjusted using a robust sandwich estimator.

^a^ Associations of maternal metabolic conditions as exposures with obstetric and neonatal complications as outcomes. Adjusted for child’s sex, birthyear, maternal age, birth order, maternal birth country, and disposable income at birth. The associations related to GDM were further adjusted for maternal BMI.

^b^ Associations of obstetric and neonatal complications as exposures with offspring NDCs as outcomes. Adjusted for child’s sex, birthyear, maternal age, birth order, maternal birth country, disposable income at birth, and maternal psychiatric history.

**Table S3.** Characteristics of the study sample over NDC outcomes.

|  | **No NDCs** | **Any NDCs** | **Autism** | **ADHD** | **ID** |
| --- | --- | --- | --- | --- | --- |
|  | **(n=2217137)** | **(n=135832)** | **(n=45654)** | **(n=101986)** | **(n=20764)** |
| **Characteristics** |  |  |  |  |  |
| **Sex** |  |  |  |  |  |
| Male | 1120771 (50.6%) | 87100 (64.1%) | 31199 (68.3%) | 65694 (64.4%) | 12519 (60.3%) |
| Female | 1096366 (49.4%) | 48732 (35.9%) | 14455 (31.7%) | 36292 (35.6%) | 8245 (39.7%) |
| **Birthyear** |  |  |  |  |  |
| 1987-1992 | 622736 (28.1%) | 35941 (26.5%) | 11622 (25.5%) | 25610 (25.1%) | 6561 (31.6%) |
| 1993-1998 | 520253 (23.5%) | 43558 (32.1%) | 14002 (30.7%) | 33453 (32.8%) | 6608 (31.8%) |
| 1999-2004 | 487584 (22.0%) | 37887 (27.9%) | 12748 (27.9%) | 30047 (29.5%) | 4680 (22.5%) |
| 2005-2010 | 586564 (26.5%) | 18446 (13.6%) | 7282 (16.0%) | 12876 (12.6%) | 2915 (14.0%) |
| **Maternal age, mean (sd)** | 29.3 (5.1) | 28.3 (5.5) | 28.9 (5.5) | 28.1 (5.5) | 28.6 (5.6) |
| **Maternal birth country** |  |  |  |  |  |
| Nordic | 1905879 (86.0%) | 120881 (89.0%) | 40167 (88.0%) | 92495 (90.7%) | 16902 (81.4%) |
| Europe | 95356 (4.3%) | 4024 (3.0%) | 1592 (3.5%) | 2535 (2.5%) | 928 (4.5%) |
| Africa | 41070 (1.9%) | 2137 (1.6%) | 762 (1.7%) | 1301 (1.3%) | 726 (3.5%) |
| Asia | 146870 (6.6%) | 6509 (4.8%) | 2317 (5.1%) | 4018 (3.9%) | 1870 (9.0%) |
| Other | 27962 (1.3%) | 2281 (1.7%) | 816 (1.8%) | 1637 (1.6%) | 338 (1.6%) |
| **Disposable income at birth** |  |  |  |  |  |
| 1(lowest) | 306819 (13.8%) | 23390 (17.2%) | 7396 (16.2%) | 16983 (16.7%) | 4840 (23.3%) |
| 2 | 448467 (20.2%) | 35187 (25.9%) | 11426 (25.0%) | 26774 (26.3%) | 5659 (27.3%) |
| 3 | 475189 (21.4%) | 30340 (22.3%) | 9978 (21.9%) | 23277 (22.8%) | 4276 (20.6%) |
| 4 | 490447 (22.1%) | 26063 (19.2%) | 8919 (19.5%) | 19809 (19.4%) | 3449 (16.6%) |
| 5(highest) | 496215 (22.4%) | 20852 (15.4%) | 7935 (17.4%) | 15143 (14.8%) | 2540 (12.2%) |
| **Birth order** |  |  |  |  |  |
| 1 | 949457 (42.8%) | 59084 (43.5%) | 21159 (46.3%) | 44188 (43.3%) | 8126 (39.1%) |
| 2 | 806705 (36.4%) | 45662 (33.6%) | 14594 (32.0%) | 34649 (34.0%) | 7044 (33.9%) |
| >=3 | 460975 (20.8%) | 31086 (22.9%) | 9901 (21.7%) | 23149 (22.7%) | 5594 (26.9%) |
| **Maternal psychiatric history** | 106613 (4.8%) | 12213 (9.0%) | 4265 (9.3%) | 9399 (9.2%) | 1907 (9.2%) |
|  |  |  |  |  |  |
| **Mediators** |  |  |  |  |  |
| **Pregnancy hypertensive diseases** | 92887 (4.2%) | 6995 (5.1%) | 2536 (5.6%) | 5058 (5.0%) | 1252 (6.0%) |
| **Placenta/membrane disorders** | 106767 (4.8%) | 8668 (6.4%) | 2852 (6.2%) | 6033 (5.9%) | 2071 (10.0%) |
| **Gestational infection** | 78887 (3.6%) | 6030 (4.4%) | 2070 (4.5%) | 4506 (4.4%) | 1027 (4.9%) |
| **Labour complications** | 74701 (3.4%) | 5042 (3.7%) | 1727 (3.8%) | 3627 (3.6%) | 870 (4.2%) |
| **Intrauterine hypoxia** | 157055 (7.1%) | 11150 (8.2%) | 3889 (8.5%) | 7855 (7.7%) | 2182 (10.5%) |
| **SGA** | 49343 (2.2%) | 5059 (3.7%) | 1682 (3.7%) | 3326 (3.3%) | 1543 (7.4%) |
| **LGA** | 76999 (3.5%) | 5177 (3.8%) | 1883 (4.1%) | 3848 (3.8%) | 784 (3.8%) |
| **Preterm birth** | 103793 (4.7%) | 9207 (6.8%) | 3075 (6.7%) | 6399 (6.3%) | 2252 (10.8%) |
| **Neonatal asphyxia comorbidities** | 90760 (4.1%) | 8682 (6.4%) | 2932 (6.4%) | 5502 (5.4%) | 2613 (12.6%) |
| **Neonatal hematological comorbidities** | 103606 (4.7%) | 8533 (6.3%) | 2935 (6.4%) | 5964 (5.8%) | 1883 (9.1%) |
| **Neonatal hypoglycemia** | 38447 (1.7%) | 3967 (2.9%) | 1439 (3.2%) | 2690 (2.6%) | 1051 (5.1%) |
| **Neonatal infection** | 72551 (3.3%) | 5788 (4.3%) | 2043 (4.5%) | 4043 (4.0%) | 1299 (6.3%) |

The characteristics of the affected and unaffected groups were compared using the Chi-squared tests for proportions. P-values were <0.001 for all covariates.

**Table S4.** Multiple mediation analysis of the association between maternal metabolic conditions and NDCs in offspring: odds ratios with 99.6% confidence intervals

**A PGDM**

|  | **Stage 1^a^** | **Stage 2^b^** | **Stage 3^c^** | **Total mediators^d^** |
| --- | --- | --- | --- | --- |
|  | **OR (95% CI)** | **OR (95% CI)** | **OR (95% CI)** | **OR (95% CI)** |
| **Any NDCs** |  |  |  |  |
| NDE | 1.37 (1.36-1.38) | 1.35 (1.34-1.36) | 1.28 (1.27-1.29) | 1.26 (1.26-1.27) |
| NIE | 1.05 (1.01-1.11) | 1.07 (1.02-1.13) | 1.13 (1.08-1.19) | 1.14 (1.09-1.21) |
| TE | 1.45 (1.38-1.53) | 1.45 (1.38-1.53) | 1.45 (1.38-1.53) | 1.44 (1.38-1.53) |
| Proportion mediated, % | 14.1** | 18.8** | 33.1** | 36.4** |
| **Autism** |  |  |  |  |
| NDE |  |  | 1.27 (1.26-1.29) | 1.23 (1.22-1.25) |
| NIE |  |  | 1.13 (1.03-1.24) | 1.17 (1.06-1.28) |
| TE |  |  | 1.44 (1.29-1.56) | 1.44 (1.29-1.56) |
| Proportion mediated, % |  |  | 34.0** | 42.9** |
| **ID** |  |  |  |  |
| NDE |  | 1.59 (1.57-1.62) | 1.33 (1.31-1.36) | 1.38 (1.35-1.40) |
| NIE |  | 1.12 (1.00-1.24) | 1.34 (1.19-1.48) | 1.29 (1.16-1.43) |
| TE |  | 1.77 (1.59-1.98) | 1.77 (1.59-1.98) | 1.77 (1.59-1.98) |
| Proportion mediated, % |  | 19.4 | 50.1** | 44.4** |
| **ADHD** |  |  |  |  |
| NDE |  |  | 1.29 (1.28-1.30) | 1.27 (1.26-1.28) |
| NIE |  |  | 1.10 (1.02-1.19) | 1.12 (1.03-1.21) |
| TE |  |  | 1.42 (1.31-1.53) | 1.42 (1.31-1.53) |
| Proportion mediated, % |  |  | 28.1** | 31.4** |

**B GDM**

|  | **Stage 1^a^** | **Stage 2^b^** | **Stage 3^c^** | **Total mediators^d^** |
| --- | --- | --- | --- | --- |
|  | **OR (95% CI)** | **OR (95% CI)** | **OR (95% CI)** | **OR (95% CI)** |
| **Any NDCs** |  |  |  |  |
| NDE |  |  |  |  |
| NIE |  |  |  |  |
| TE |  |  |  |  |
| Proportion mediated, % |  |  |  |  |
| **Autism** |  |  |  |  |
| NDE |  |  |  |  |
| NIE |  |  |  |  |
| TE |  |  |  |  |
| Proportion mediated, % |  |  |  |  |
| **ID** |  |  |  |  |
| NDE |  |  | 1.21 (1.19-1.23) |  |
| NIE |  |  | 1.12 (0.96-1.35) |  |
| TE |  |  | 1.37 (1.15-1.62) |  |
| Proportion mediated, % |  |  | 37.6 |  |
| **ADHD** |  |  |  |  |
| NDE |  |  |  |  |
| NIE |  |  |  |  |
| TE |  |  |  |  |
| Proportion mediated, % |  |  |  |  |

**C Adiposity**

|  | **Stage 1^a^** | **Stage 2^b^** | **Stage 3^c^** | **Total mediators^d^** |
| --- | --- | --- | --- | --- |
|  | **OR (95% CI)** | **OR (95% CI)** | **OR (95% CI)** | **OR (95% CI)** |
| **Any NDCs** |  |  |  |  |
| NDE |  |  |  | 1.34 (1.33-1.36) |
| NIE |  |  |  | 1.01 (1.00-1.02) |
| TE |  |  |  | 1.36 (1.34-1.38) |
| Proportion mediated, % |  |  |  | 3.6 |
| **Autism** |  |  |  |  |
| NDE |  |  |  | 1.34 (1.32-1.37) |
| NIE |  |  |  | 1.02 (1.00-1.04) |
| TE |  |  |  | 1.36 (1.33-1.40) |
| Proportion mediated, % |  |  |  | 5.3 |
| **ID** |  |  |  |  |
| NDE |  |  | 1.53 (1.48-1.58) | 1.52 (1.47-1.56) |
| NIE |  |  | 1.03 (0.99-1.06) | 1.04 (1.01-1.07) |
| TE |  |  | 1.57 (1.50-1.63) | 1.58 (1.51-1.64) |
| Proportion mediated, % |  |  | 5.5 | 8.1** |
| **ADHD** |  |  |  |  |
| NDE |  |  |  |  |
| NIE |  |  |  |  |
| TE |  |  |  |  |
| Proportion mediated, % |  |  |  |  |

A replication of the multiple mediation analysis used a 99.6% confidence interval when the 95% confidence interval for NIEs in Table 3 and Table 4 did not encompass a value of 1. Blank areas in the tables indicated that the 95% confidence interval encompassed the value of 1; as a result, we did not replicate these models using a 99.6% confidence interval.

All analyses were adjusted for the child’s sex, birth year, maternal age, birth order, maternal birth country, disposable income at birth, and maternal psychiatric history. Analyses for GDM also included an adjustment for maternal BMI. Proportions mediated were marked with “**” when the 99.6% confidence interval for NIE did not encompass a value of 1, with NDE and NIE pointing in the same direction.

^a^ Mediators in stage 1 for PGDM and GDM included: pregnancy hypertensive diseases, placenta/membrane disorders, and gestational infection.

Mediators in stage 1 for adiposity include: pregnancy hypertensive diseases, placenta/membrane disorders, gestational infection, and GDM.

^b^ Mediators in stage 2 included: SGA, and LGA, intrauterine hypoxia, labour complications, and preterm birth.

^c^ Mediators in stage 3 included: neonatal asphyxia comorbidities, neonatal hematological comorbidities, neonatal hypoglycemia, and neonatal infection.

^d^ This included all mediators from stages 1, 2, and 3.

**Table S5.** Sensitivity analysis for the single mediation analysis in the association between T1DM and any NDCs.

|  | **T1DM** |  |
| --- | --- | --- |
| **Pregnancy hypertensive diseases** | **OR (95% CI)** | **P-value** |
| NDE | 1.36 (1.28-1.44) | <0.001 |
| NIE | 1.04 (1.02-1.06) | <0.001 |
| TE | 1.41 (1.34-1.49) | <0.001 |
| Prop.mediated, % | 10.52** |  |
| **Placenta/membrane disorders** |  |  |
| NDE | 1.40 (1.33-1.48) | <0.001 |
| NIE | 1.01 (1.00-1.01) | 0.011 |
| TE | 1.41 (1.34-1.49) | <0.004 |
| Prop.mediated, % | 2.20* |  |
| **Gestational infection** |  |  |
| NDE | 1.40 (1.33-1.48) | <0.001 |
| NIE | 1.01 (1.00-1.02) | 0.055 |
| TE | 1.41 (1.34-1.49) | <0.001 |
| Prop.mediated, % | 2.32 |  |
| **Labour complications** |  |  |
| NDE | 1.41 (1.33-1.48) | <0.001 |
| NIE | 1.00 (1.00-1.01) | 0.339 |
| TE | 1.41 (1.34-1.49) | <0.001 |
| Prop.mediated, % | 0.91 |  |
| **Intrauterine hypoxia** |  |  |
| NDE | 1.39 (1.31-1.46) | <0.001 |
| NIE | 1.01 (1.00-1.03) | 0.009 |
| TE | 1.41 (1.34-1.49) | <0.001 |
| Prop.mediated, % | 4.34* |  |
| **SGA** |  |  |
| NDE | 1.39 (1.29-1.47) | <0.001 |
| NIE | 1.00 (1.00-1.00) | 0.180 |
| TE | 1.39 (1.29-1.47) | <0.001 |
| Prop.mediated, % | 0.33 |  |
| **LGA** |  |  |
| NDE | 1.40 (1.31-1.49) | <0.001 |
| NIE | 1.01 (0.99-1.04) | 0.451 |
| TE | 1.41 (1.32-1.50) | <0.001 |
| Prop.mediated, % | 2.97 |  |
| **Preterm birth** |  |  |
| NDE | 1.34 (1.26-1.42) | <0.001 |
| NIE | 1.05 (1.03-1.07) | <0.001 |
| TE | 1.41 (1.33-1.49) | <0.001 |
| Prop.mediated, % | 15.39** |  |
| **Neonatal asphyxia comorbidities** |  |  |
| NDE | 1.35 (1.28-1.43) | <0.001 |
| NIE | 1.04 (1.02-1.06) | <0.001 |
| TE | 1.41 (1.34-1.49) | <0.001 |
| Prop.mediated, % | 12.04** |  |
| **Neonatal hematological comorbidities** |  |  |
| NDE | 1.37 (1.29-1.45) | <0.001 |
| NIE | 1.03 (1.01-1.04) | 0.001 |
| TE | 1.41 (1.34-1.49) | <0.001 |
| Prop.mediated, % | 7.88** |  |
| **Neonatal hypoglycemia** |  |  |
| NDE | 1.36 (1.29-1.44) | <0.001 |
| NIE | 1.03 (1.01-1.06) | 0.006 |
| TE | 1.41 (1.34-1.49) | <0.001 |
| Prop.mediated, % | 9.50 |  |
| **Neonatal infection** |  |  |
| NDE | 1.39 (1.31-1.47) | <0.001 |
| NIE | 1.02 (1.01-1.03) | <0.001 |
| TE | 1.41 (1.34-1.49) | <0.001 |
| Prop.mediated, % | 5.60** |  |

All analyses were adjusted for the child’s sex, birth year, maternal age, birth order, maternal birth country, disposable income at birth, and maternal psychiatric history. Proportions mediated were marked with “*” when P < 0.05 for NIE and with “**” when P_Bonferroni corrected_ < 0.004 for NIE, with both NDE and NIE having the same direction.

**Table S6.** Sensitivity analysis for the single mediation analysis of the association between maternal adverse metabolic conditions and any NDCs among offspring born no earlier than 1997.

|  | **PGDM** |  | **GDM** |  | **Adiposity** |  |
| --- | --- | --- | --- | --- | --- | --- |
| **Pregnancy hypertensive diseases** | **OR (95% CI)** | **P-value** | **OR (95% CI)** | **P-value** | **OR (95% CI)** | **P-value** |
| NDE | 1.44 (1.33-1.55) | <0.001 | 1.09 (1.00-1.17) | 0.036 | 1.39 (1.36-1.41) | <0.001 |
| NIE | 1.05 (1.02-1.07) | <0.001 | 1.00 (1.00-1.01) | 0.553 | 1.01 (1.01-1.01) | <0.001 |
| TE | 1.51 (1.41-1.63) | <0.001 | 1.09 (1.01-1.17) | 0.028 | 1.40 (1.37-1.42) | <0.001 |
| Prop.mediated, % | 11.36** |  | 2.63 |  | 2.22** |  |
| **Placenta/membrane disorders** |  |  |  |  |  |  |
| NDE | 1.49 (1.39-1.61) | <0.001 | 1.09 (1.01-1.17) | 0.031 | 1.40 (1.38-1.43) | <0.001 |
| NIE | 1.01 (1.01-1.02) | 0.002 | 1.00 (1.00-1.01) | 0.627 | 1.00 (1.00-1.00) | <0.001 |
| TE | 1.51 (1.41-1.63) | <0.001 | 1.09 (1.01-1.17) | 0.028 | 1.40 (1.38-1.42) | <0.001 |
| Prop.mediated, % | 3.36** |  | 1.36 |  | -0.31 |  |
| **Gestational infection** | |  |  |  |  |  |
| NDE | 1.48 (1.39-1.60) | <0.001 | 1.09 (1.01-1.17) | 0.033 | 1.40 (1.37-1.42) | <0.001 |
| NIE | 1.02 (1.00-1.03) | 0.013 | 1.00 (1.00-1.01) | 0.273 | 1.00 (1.00-1.00) | <0.001 |
| TE | 1.51 (1.41-1.63) | <0.001 | 1.09 (1.01-1.17) | 0.029 | 1.40 (1.38-1.42) | <0.001 |
| Prop.mediated, % | 3.66* |  | 2.33 |  | 0.66** |  |
| **Labour complications** | |  |  |  |  |  |
| NDE | 1.50 (1.40-1.62) | <0.001 | 1.09 (1.00-1.17) | 0.042 | 1.40 (1.38-1.42) | <0.001 |
| NIE | 1.00 (1.00-1.01) | 0.215 | 1.00 (1.00 -1.00) | 0.824 | 1.00 (1.00-1.00) | 0.172 |
| TE | 1.50 (1.41-1.63) | <0.001 | 1.09 (1.01-1.17) | 0.027 | 1.40 (1.38-1.42) | <0.001 |
| Prop.mediated, % | 1.22 |  | -0.23 |  | 0.09 |  |
| **Intrauterine hypoxia** | |  |  |  |  |  |
| NDE | 1.49 (1.38-1.61) | <0.001 | 1.08 (1.00-1.17) | 0.042 | 1.40 (1.37-1.42) | <0.001 |
| NIE | 1.01 (1.00-1.03) | 0.066 | 1.00 (1.00-1.00) | 0.207 | 1.00 (1.00-1.00) | <0.001 |
| TE | 1.50 (1.41-1.63) | <0.001 | 1.08 (1.00-1.17) | 0.039 | 1.40 (1.37-1.42) | <0.001 |
| Prop.mediated, % | 3.02 |  | 1.47 |  | 0.93** |  |
| **SGA** |  |  |  |  |  |  |
| NDE | 1.47 (1.35-1.59) | <0.001 | 1.06 (0.98-1.16) | 0.170 | 1.40 (1.37-1.42) | <0.001 |
| NIE | 1.00 (1.00-1.00) | 0.757 | 1.00 (0.99-1.00) | 0.141 | 1.00 (1.00-1.00) | <0.001 |
| TE | 1.47 (1.35-1.59) | <0.001 | 1.06 (0.97-1.15) | 0.189 | 1.40 (1.37-1.42) | <0.001 |
| Prop.mediated, % | 0.08 |  | -4.43 |  | -0.21 |  |
| **LGA** |  |  |  |  |  |  |
| NDE | 1.47 (1.37-1.60) | <0.001 | 1.08 (0.98-1.17) | 0.103 | 1.40 (1.37-1.42) | <0.001 |
| NIE | 1.02 (0.99-1.06) | 0.217 | 1.02 (1.00-1.03) | 0.039 | 1.00 (1.00-1.00) | 0.076 |
| TE | 1.50 (1.40-1.61) | <0.001 | 1.09 (1.00-1.18) | 0.038 | 1.40 (1.38-1.43) | <0.001 |
| Prop.mediated, % | 5.24 |  | 17.68* |  | 0.47 |  |
| **Preterm birth** | |  |  |  |  |  |
| NDE | 1.41 (1.31-1.52) | <0.001 | 1.10 (1.01-1.18) | 0.019 | 1.40 (1.37-1.42) | <0.001 |
| NIE | 1.07 (1.05-1.11) | <0.001 | 1.01 (1.00-1.02) | 0.091 | 1.00 (1.00-1.00) | <0.001 |
| TE | 1.51 (1.42-1.62) | <0.001 | 1.11 (1.02-1.19) | 0.009 | 1.40 (1.38-1.43) | <0.001 |
| Prop.mediated, % | 17.39** |  | 8.41 |  | 1.19** |  |
| **Neonatal asphyxia comorbidities** |  |  |  |  |  |  |
| NDE | 1.44 (1.34-1.55) | <0.001 | 1.08 (1.00-1.16) | 0.060 | 1.39 (1.37-1.42) | <0.001 |
| NIE | 1.04 (1.02-1.06) | <0.001 | 1.01 (1.00-1.01) | 0.009 | 1.01 (1.00-1.01) | <0.001 |
| TE | 1.50 (1.40-1.62) | <0.001 | 1.09 (1.00-1.17) | 0.037 | 1.40 (1.38-1.42) | <0.001 |
| Prop.mediated, % | 10.72** |  | 9.28* |  | 1.70** |  |
| **Neonatal hematological comorbidities** |  |  |  |  |  |  |
| NDE | 1.44 (1.35-1.55) | <0.001 | 1.09 (1.01-1.17) | 0.030 | 1.40 (1.37-1.42) | <0.001 |
| NIE | 1.04 (1.02-1.06) | <0.001 | 1.00 (1.00-1.01) | 0.513 | 1.00 (1.00-1.01) | <0.001 |
| TE | 1.50 (1.40-1.62) | <0.001 | 1.09 (1.01-1.17) | 0.026 | 1.40 (1.38-1.42) | <0.001 |
| Prop.mediated, % | 9.81** |  | 1.92 |  | 1.21** |  |
| **Neonatal hypoglycemia** |  |  |  |  |  |  |
| NDE | 1.46 (1.36-1.60) | <0.001 | 1.07 (0.99-1.16) | 0.111 | 1.39 (1.37-1.41) | <0.001 |
| NIE | 1.03 (1.00-1.06) | 0.058 | 1.02 (1.00-1.04) | 0.051 | 1.01 (1.00-1.01) | <0.001 |
| TE | 1.51 (1.41-1.63) | <0.001 | 1.09 (1.01-1.17) | 0.035 | 1.40 (1.37-1.42) | <0.001 |
| Prop.mediated, % | 7.23 |  | 20.77 |  | 1.77** |  |
| **Neonatal infection** | |  |  |  |  |  |
| NDE | 1.47 (1.38-1.60) | <0.001 | 1.08 (1.00-1.16) | 0.042 | 1.40 (1.37-1.42) | <0.001 |
| NIE | 1.02 (1.01-1.04) | <0.001 | 1.00 (1.00-1.01) | 0.079 | 1.00 (1.00-1.00) | <0.001 |
| TE | 1.51 (1.41-1.63) | <0.001 | 1.09 (1.00-1.17) | 0.032 | 1.40 (1.38-1.42) | <0.001 |
| Prop.mediated, % | 5.77** |  | 4.84 |  | 0.62** |  |
| **GDM** |  |  |  |  |  |  |
| NDE |  |  |  |  | 1.39 (1.37-1.42) | <0.001 |
| NIE |  |  |  |  | 1.00 (1.00-1.00) | <0.001 |
| TE |  |  |  |  | 1.40 (1.37-1.42) | <0.001 |
| Prop.mediated, % |  |  |  |  | 0.68** |  |

All analyses were adjusted for child’s sex, birthyear, maternal age, birth order, maternal birth country, disposable income at birth, maternal

psychiatric history. Analyses for GDM were additionally adjusted for maternal BMI. Proportions mediated were marked with “*” when P <0.05 for NIE and with “**” when P_Bonferroni corrected_ <0.004 for NIE, with both NDE and NIE having the same direction.

**Table S7.** Sensitivity analysis for the single mediation analysis of the association between maternal metabolic conditions and any NDCs, derived by randomly selecting one child from each mother.

|  | **PGDM** |  | **GDM** |  | **Adiposity** |  |
| --- | --- | --- | --- | --- | --- | --- |
| **Pregnancy hypertensive diseases** | **OR (95% CI)** | **P-value** | **OR (95% CI)** | **P-value** | **OR (95% CI)** | **P-value** |
| NDE | 1.35 (1.26-1.45) | <0.001 | 1.23 (1.14-1.32) | <0.001 | 1.39 (1.36-1.41) | <0.001 |
| NIE | 1.05 (1.02-1.07) | <0.001 | 1.01 (1.00-1.02) | 0.077 | 1.01 (1.00-1.01) | <0.001 |
| TE | 1.41 (1.33-1.51) | <0.001 | 1.24 (1.15-1.32) | <0.001 | 1.39 (1.37-1.42) | <0.001 |
| Prop.mediated, % | 13.19** |  | 3.36 |  | 1.94** |  |
| **Placenta/membrane disorders** |  |  |  |  |  |  |
| NDE | 1.40 (1.32-1.50) | <0.001 | 1.24 (1.16-1.33) | <0.001 | 1.40 (1.37-1.42) | <0.001 |
| NIE | 1.01 (1.00-1.02) | 0.002 | 1.00 (1.00-1.01) | 0.346 | 1.00 (1.00-1.00) | <0.001 |
| TE | 1.42 (1.33-1.52) | <0.001 | 1.24 (1.16-1.33) | <0.001 | 1.40 (1.37-1.42) | <0.001 |
| Prop.mediated, % | 3.14** |  | 0.85 |  | -0.29 |  |
| **Gestational infection** |  |  |  |  |  |  |
| NDE | 1.40 (1.32-1.51) | <0.001 | 1.25 (1.16-1.34) | <0.001 | 1.39 (1.37-1.42) | <0.001 |
| NIE | 1.01 (1.00-1.02) | 0.099 | 1.00 (1.00-1.00) | 0.723 | 1.00 (1.00-1.00) | <0.001 |
| TE | 1.42 (1.33-1.52) | <0.001 | 1.25 (1.16-1.33) | <0.001 | 1.40 (1.37-1.42) | <0.001 |
| Prop.mediated, % | 2.60 |  | -0.25 |  | 0.57** |  |
| **Labour complications** |  |  |  |  |  |  |
| NDE | 1.41 (1.33-1.51) | <0.001 | 1.24 (1.15-1.33) | <0.001 | 1.40 (1.37-1.42) | <0.001 |
| NIE | 1.00 (1.00-1.01) | 0.263 | 1.00 (1.00-1.00) | 0.242 | 1.00 (1.00-1.00) | 0.506 |
| TE | 1.42 (1.33-1.52) | <0.001 | 1.24 (1.16-1.33) | <0.001 | 1.40 (1.37-1.42) | <0.001 |
| Prop.mediated, % | 1.14 |  | 0.69 |  | 0.05 |  |
| **Intrauterine hypoxia** |  |  |  |  |  |  |
| NDE | 1.40 (1.31-1.49) | <0.001 | 1.24 (1.16-1.33) | <0.001 | 1.39 (1.36-1.42) | <0.001 |
| NIE | 1.01 (1.00-1.02) | 0.062 | 1.00 (1.00-1.00) | 0.426 | 1.00 (1.00-1.00) | <0.001 |
| TE | 1.41 (1.33-1.51) | <0.001 | 1.24 (1.16-1.34) | <0.001 | 1.39 (1.37-1.42) | <0.001 |
| Prop.mediated, % | 3.31 |  | 0.16 |  | 0.80** |  |
| **SGA** |  |  |  |  |  |  |
| NDE | 1.41 (1.30-1.52) | <0.001 | 1.24 (1.15-1.35) | <0.001 | 1.40 (1.37-1.43) | <0.001 |
| NIE | 1.00 (1.00-1.01) | 0.085 | 1.00 (0.99-1.00) | 0.045 | 1.00 (1.00-1.00) | 0.002 |
| TE | 1.42 (1.30-1.52) | <0.001 | 1.24 (1.15-1.34) | <0.001 | 1.40 (1.37-1.43) | <0.001 |
| Prop.mediated, % | 0.85 |  | -1.73 |  | -0.22 |  |
| **LGA** |  |  |  |  |  |  |
| NDE | 1.40 (1.29-1.50) | <0.001 | 1.24 (1.15-1.33) | <0.001 | 1.40 (1.37-1.42) | <0.001 |
| NIE | 1.00 (0.97-1.03) | 0.873 | 1.01 (0.99-1.02) | 0.406 | 1.00 (1.00-1.00) | 0.758 |
| TE | 1.41 (1.30-1.51) | <0.001 | 1.25 (1.16-1.34) | <0.001 | 1.40 (1.37-1.42) | <0.001 |
| Prop.mediated, % | 0.65 |  | 2.28 |  | 0.09 |  |
| **Preterm birth** |  |  |  |  |  |  |
| NDE | 1.35 (1.25-1.44) | <0.001 | 1.24 (1.15-1.33) | <0.001 | 1.39 (1.36-1.42) | <0.001 |
| NIE | 1.06 (1.03-1.08) | <0.001 | 1.00 (1.00-1.01) | 0.484 | 1.00 (1.00-1.00) | <0.001 |
| TE | 1.43 (1.34-1.52) | <0.001 | 1.24 (1.15-1.32) | <0.001 | 1.40 (1.36-1.42) | <0.001 |
| Prop.mediated, % | 15.67** |  | 1.18 |  | 1.10** |  |
| **Neonatal asphyxia comorbidities** |  |  |  |  |  |  |
| NDE | 1.35 (1.27-1.44) | <0.001 | 1.24 (1.15-1.32) | <0.001 | 1.39 (1.36-1.41) | <0.001 |
| NIE | 1.04 (1.03-1.06) | <0.001 | 1.01 (1.00-1.01) | 0.020 | 1.01 (1.01-1.01) | <0.001 |
| TE | 1.41 (1.33-1.51) | <0.001 | 1.24 (1.16-1.33) | <0.001 | 1.40 (1.37-1.42) | <0.001 |
| Prop.mediated, % | 12.43** |  | 2.61* |  | 1.86** |  |
| **Neonatal hematological comorbidities** |  | | | | |  |
| NDE | 1.37 (1.28-1.47) | <0.001 | 1.24 (1.16-1.33) | <0.001 | 1.39 (1.36-1.42) | <0.001 |
| NIE | 1.03 (1.01-1.05) | 0.001 | 1.00 (1.00-1.01) | 0.316 | 1.00 (1.00-1.01) | <0.001 |
| TE | 1.41 (1.33-1.51) | <0.001 | 1.25 (1.16-1.33) | <0.001 | 1.40 (1.37-1.42) | <0.001 |
| Prop.mediated, % | 8.30** |  | 1.31 |  | 1.26** |  |
| **Neonatal hypoglycemia** |  |  |  |  |  |  |
| NDE | 1.37 (1.29-1.48) | <0.001 | 1.24 (1.15-1.33) | <0.001 | 1.39 (1.36-1.42) | <0.001 |
| NIE | 1.03 (1.00-1.06) | 0.035 | 1.00 (1.00-1.02) | 0.284 | 1.00 (1.00-1.01) | <0.001 |
| TE | 1.41 (1.33-1.51) | <0.001 | 1.24 (1.16-1.34) | <0.001 | 1.39 (1.37-1.42) | <0.001 |
| Prop.mediated, % | 7.98* |  | 2.70 |  | 1.28** |  |
| **Neonatal infection** |  |  |  |  |  |  |
| NDE | 1.40 (1.32-1.49) | <0.001 | 1.24 (1.15-1.33) | <0.001 | 1.39 (1.37-1.42) | <0.001 |
| NIE | 1.01 (1.01-1.02) | 0.002 | 1.00 (1.00-1.00) | 0.239 | 1.00 (1.00-1.00) | <0.001 |
| TE | 1.42 (1.33-1.52) | <0.001 | 1.24 (1.16-1.33) | <0.001 | 1.40 (1.37-1.42) | <0.001 |
| Prop.mediated, % | 4.19** |  | 0.65 |  | 0.62** |  |
| **GDM** |  |  |  |  |  |  |
| NDE |  |  |  |  | 1.39 (1.35-1.41) | <0.001 |
| NIE |  |  |  |  | 1.00 (1.00-1.00) | <0.001 |
| TE |  |  |  |  | 1.39 (1.36-1.42) | <0.001 |
| Prop.mediated, % |  |  |  |  | 0.94** |  |

All analyses were adjusted for child’s sex, birthyear, maternal age, birth order, maternal birth country, disposable income at birth, maternal

psychiatric history. Analyses for GDM were additionally adjusted for maternal BMI. Proportions mediated were marked with “*” when P <0.05 for NIE and with “**” when P_Bonferroni corrected_ <0.004 for NIE, with both NDE and NIE having the same direction.
